# Advances in Research on Chemical Constituents and Their Biological Activities of the Genus *Actinidia*

**DOI:** 10.1007/s13659-021-00319-8

**Published:** 2021-09-30

**Authors:** Jin-Tao Ma, Da-Wei Li, Ji-Kai Liu, Juan He

**Affiliations:** 1 School of Pharmaceutical Sciences, National Demonstration Center for Experimental Ethnopharmacology Education, South-Central University for Nationalities, Wuhan, 430074 People’s Republic of China; 2 Center of Economic Botany, Core Botanical Gardens, Chinese Academy of Sciences, Wuhan, 430074 People’s Republic of China

**Keywords:** *Actinidia* chemical constituents, Isolation, Biological activities

## Abstract

Kiwi, a fruit from plants of the genus *Actinidia*, is one of the famous fruits with thousand years of edible history. In the past twenty years, a great deal of research has been done on the chemical constituents of the *Actinidia* species. A large number of secondary metabolites including triterpenoids, flavonoids, phenols, etc. have been identified from differents parts of *Actinidia* plants, which exhibited significant in vitro and in vivo pharmacological activities including anticancer, anti-inflammatory, neuroprotective, anti-oxidative, anti-bacterial, and anti-diabetic activities. In order to fully understand the chemical components and biological activities of *Actinidia* plants, and to improve their further research, development and utilization, this review summarizes the compounds extracted from different parts of *Actinidia* plants since 1959 to 2020, classifies the types of constituents, reports on the pharmacological activities of relative compounds and medicinal potentials.

## Introduction

**Fig. 1:**
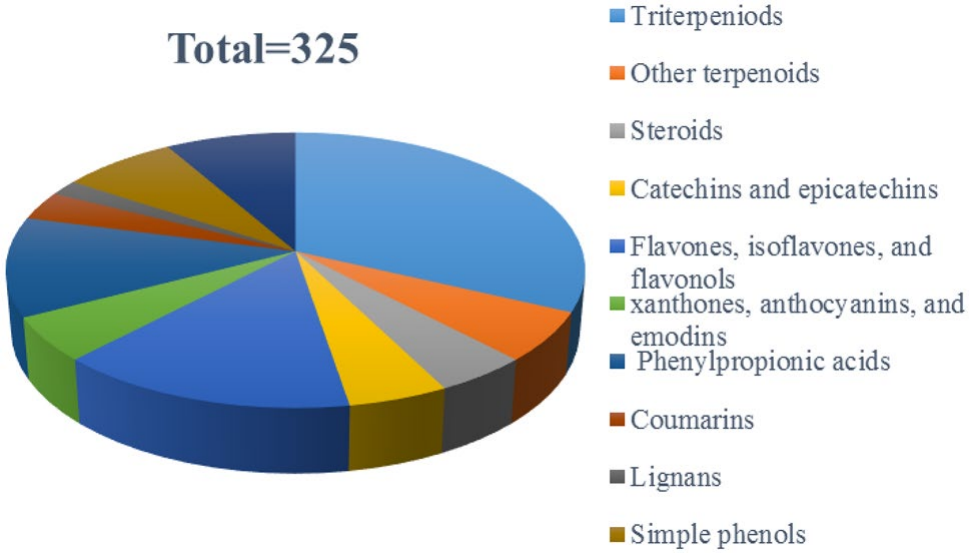
Constituents proportion of 12 *Actinidia* plants

With the development of natural product research, a huge number of chemical constituents have been identified from natural resources. There is no doubt that the research on the chemical composition of fruits, including trace elements, has greatly improved the application prospects of these fruits. With no exception, it is the same to kiwifruit, one of the most prestigious fruits with a long history of eating [1, 2]. Kiwi belongs to plants of the genus *Actinidia* comprising more than 70 species around the world [3]. Some of these plants are proven to have a wide range of medicinal activities. For example, *A. valvata*, whose root is known as ‘‘Mao-Ren-Shen” in traditional Chinese medicine, exhibits antitumor and anti-inflammatory activities and has been used for the treatment of hepatoma, lung carcinoma and myeloma for a long time [4, 5]. The roots of *A. chinensis* Planch, called “Teng-Li-Gen” usually, were used as a traditional Chinese medicine for the treatment of various cancers, such as esophagus cancer, liver cancer, and gastric cancer [6]. In the past two decades, great research had been accomplished about exploring the chemical composition of *Actinidia* plants. These studies have greatly promoted the understanding of the chemical components and functions of the *Actinidia* plant. According to literature survey, 12 *Actinidia* species including *A. valvata*, *A. chinensis, A. argute*, *A. polygama*, *A. kolomikta*, *A. eriantha*, *A. macrosperma*, *A. deliciosa*, *A. chrysantha*, *A. rufa*, *A. indochinensis*, and *A. valvata* were reported for their natural products. This review systematically summarizes the chemical components and their biological activities from different parts of 12 *Actinidia* species from 1959 to 2020. According to structure types, a total of 325 molecules have been collected including terpeniods, phenols, and other small groups (Fig. 1). Names and isolation information were listed in the tables, while the biological activities of the extracts or compounds were discussed in the text.

## Chemical Constituents

### Terpenoids

In recent years, a large number of terpenoids were isolated from many *Actinidia* species. Among them, triterpenes account for the vast majority that are mainly composed by several normal frameworks including ursane-type, oleanane-type, and lupane-type. Of the total 325 compounds in this review, 104 are triterpenoids. From the literature review, ursolic acids and their saponins are undoubtedly the most abundant in *Actinidia* species.

#### Ursane Triterpenoids

**Fig. 2:**
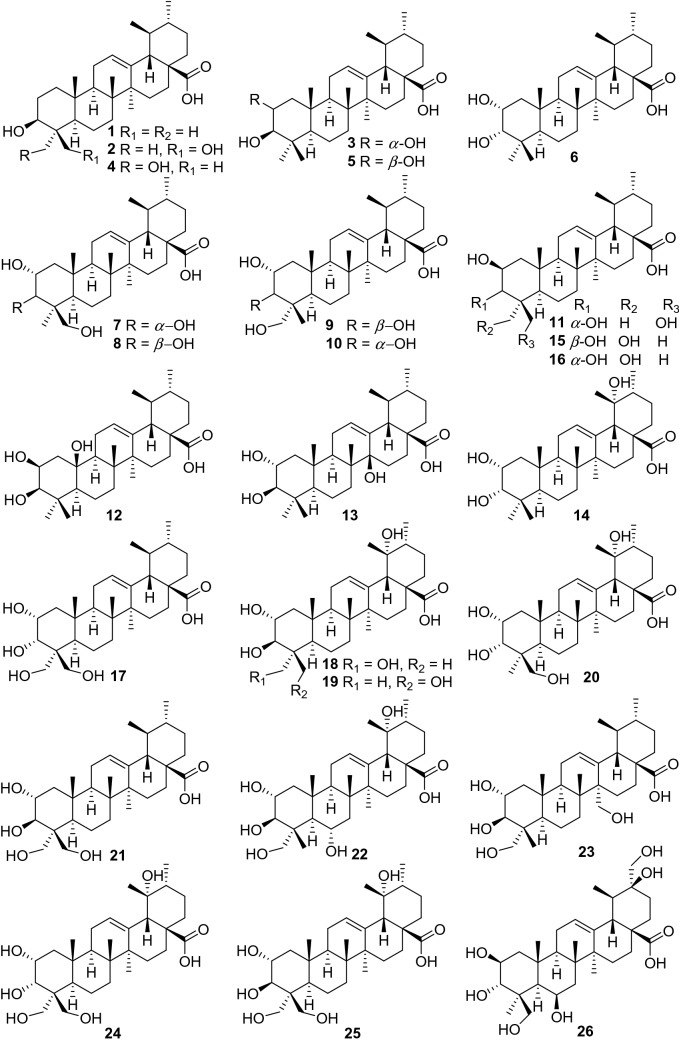

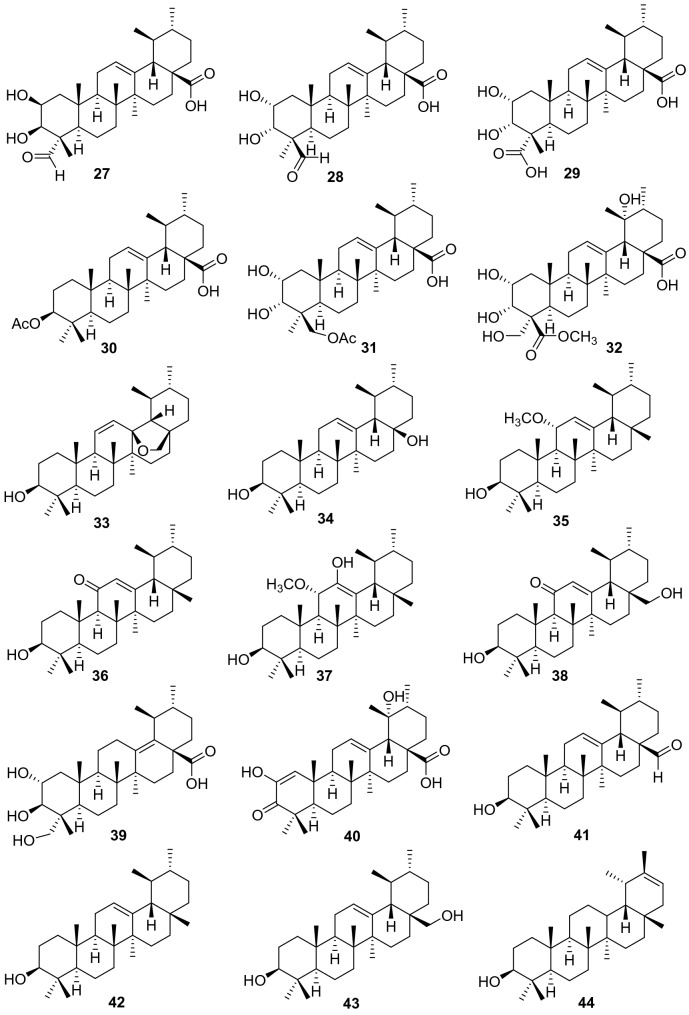

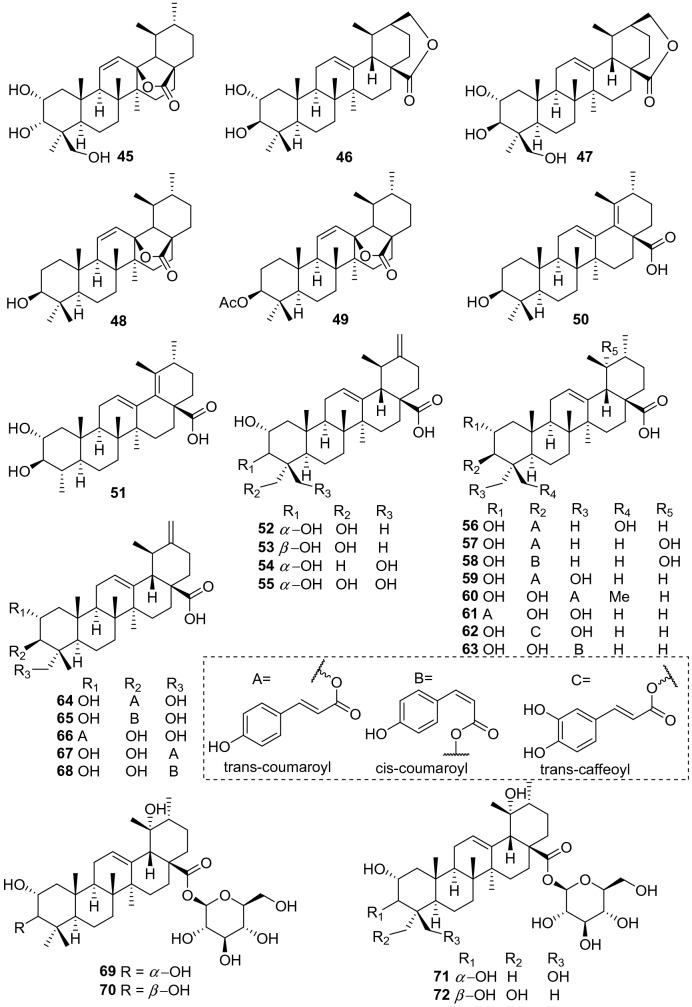

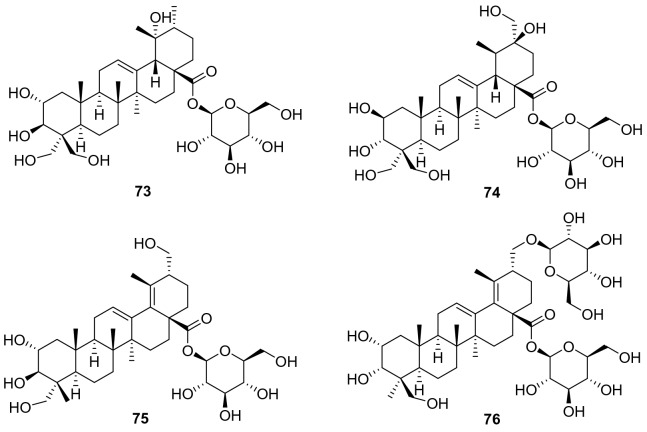
Structures of ursane triterpenoids **1**‒**76** from *Actinidia* plants

Ursane-type triterpenes are characterized of ursolic acid and its saponins, possessing a 6/6/6/6/6-fused carbon skeleton. A total of 76 ursane-type triterpenoids (**1**–**76**) have been identified from plants of the genus *Actinidia* (Fig. 2, Table 1). Ursolic acid (3*β*-Hydroxyurs-12-en-28-oic acid **1)** [7], is one of the most frequently obtained compound in many kinds of kiwifruit plants with unique flavor. Great attention had been paid on biological activities about ursolic acid, attracting much interest in recent years. Ursolic acid exhibits different pharmacological activities, including anti-cancer, amylolytic enzyme inhibitors, cytotoxicity, downregulating thymic stromal lymphopoietin and others [7–11].

**Table 1:** Information of ursane triterpenoids from *Actinidia* plants

No.	Compound name	Species Refs.	Part	Bioactivity Refs.
**1**	3*β*-Hydroxyurs-12-en-28-oic acid	*A. eriantha* [7]	Roots	Anticancer (in vitro) [8] Inhibiting amylolytic enzyme (in vitro) [9] Antidepressant and neuroprotective (in vitro) [10] Downregulating thymic stromal lymphopoietin (in vitro) [11]
**2**	3*β*,24-Dihydroxyurs-12-en-28-oic acid	*A. arguta* [37] *A. polygama* [23]	Leaves Fruit galls	
**3**	2*α*,3*β*-Dihydroxyurs-12-en-28-oic acid	*A. polygama* [23] *A. arguta* [13]	Fruit galls Roots	Anti-metastation (In vitro) [38] Anticancer (In vitro) [12]
**4**	23-Hydroxyursolic acid	*A. arguta* [13]	Roots	Anti-pancreatic lipase (In vitro) [13] Anticancer(In vitro) [14–16] Anti-inflammatory (in vitro) [17]
**5**	2*β*,3*β*-Dihydroxyursolic acid	*A.* sp [39]		
**6**	2*α*,3*α*-Dihydroxyurs-12-en-28-oic acid	*A. chinensis* [30]	Roots	
**7**	2*α*,3*α*,24-Trihydroxyurs-12-en-28-oic acid	*A. eriantha* [40]	Roots	
**8**	2*α*,3*β*,24-Trihydroxy-urs-12-en-28-oic acid	*A. eriantha* Benth [41] *A. polygama* [18]	Fruit galls	Against A549, LOVO, and HepG2 cell lines (in vitro) [19]
**9**	2*α*,3*β*,23-Trihydroxyurs-12-en-28-oic acid	*A. polygama* [18] *A. chrysantha* [42]	Fruit galls Roots	Against A549, LOVO, and HepG2 cell lines (in vitro) [19]
**10**	2*α*,3*α*,23-Trihydroxyurs-12-en-28-oic acid	*A. polygama* [18]	Fruit galls	
**11**	2*β*,3*α*,24-Trihydroxy-urs-12-en-28-oic acid	*A. rufa* [43]	Roots	
**12**	2*β*,3*β*,10-Trihydroxy ursolic acid	*A.* sp [39]		
**13**	2*α*,3*β*,14*β*-Trihydroxy ursolic acid	*A.* sp [39]		
**14**	2*α*,3*α*,19-Trihydroxyurs-12-en-28-oic acid	*A*. *chinensis* [44]	Roots	Inhibiting tumor angiogenesis (in vitro) [45]
**15**	2*β*,3*β*,23-Trihydroxyurs-12-en-28-oic acid	*A. eriantha* [7]	Roots	Against HepG2, A549, MCF-7, SK-OV-3, and HeLa cell lines (in vitro) [32]
**16**	2*β*,3*α*,23-Trihydroxyurs-12-en-28-oic acid	*A*. *chinensis* Planch [32]	Roots	Against HepG2, A549, MCF-7, SK-OV-3, and HeLa cell lines (in vitro) [32]
**17**	2*α*,3*α*,23,24-Tetrahydroxyurs-12-en-28-oic acid	*A. polygama* [18]	Fruit galls	Against A549, LOVO and HepG2 cell lines (in vitro) [20]
**18**	2*α*, 3*β*, 19*α*, 23-Tetrahydroxyurs-12-en-28-oic acid	*A. chinensis* Planch [32]	Roots	
**19**	2*α*,3*β*,19*α*,24-Tetrahydroxyurs-12-en-28-oic acid	*A. indochinensis* Merr. Var [46]	Roots	
**20**	2*α*,3*α*,19*α*,24-Tetrahydroxyurs-12-en-28-oic acid	*A. valvata* [21]	Leaves	Anti-tumor (in vitro) [21]
**21**	2*α*,3*β*,23,24-Tetrahydroxyurs-12-en-28-oic acid	*A. chinensis* Planch [32]	Roots	
**22**	2*α*,3*β*,6*β*,23-Tetrahydroxyurs-12-en-28-oic acid	*A. valvata* Dunn [47]	Roots	
*23*	2*α*,3*β*,23,27-Tetrahydroxy-12-en-28-ursolic acid	*A*. *deliciosa* [48]	Roots	
**24**	2*α*,3*α*,19*α*,23,24-Pentahydroxy-urs-12-en- 28-oic acid	*A. chinensis* Planch [19]	Roots	
**25**	2*α*,3*β*,19*α*,23,24-Pentahydroxyurs-12-en-28-oic acid	*A. rufa* Planch ex miq [43]	Roots	
**26**	(2*β*,3*α*,6*α*)-2,3,6,20,23,30-Hexahydroxyurs-12-en-28-oic acid	*A. valvata* Dunn [22]	Roots	Against BEL-7402 and SMMC-7721 cells lines (in vitro) [22]
**27**	2*β*,3*β*-Dihydroxy-23-oxours-12-en-28-oic acid	*A. eriantha* Benth [49]	Roots	
**28**	2*α*,3*α*-Dihydroxyurs-12-ene-24-al-28-oic acid	*A. polygama* [18]	Fruit galls	
**29**	2*α*,3*α*,24-Trihydroxyurs-12-ene-23,28-dioic acid	*A. polygama* [18]	Fruit galls	
**30**	3*β*-O-Acetylursolic acid	*A. polygama* [23]	Fruit galls	Inhibiting PTP1B (in vitro) [24]
**31**	24-Acetyloxy-2*α*,3*α*-dihydroxyurs-12-en-28-oic acid	*A. eriantha* [7]	Roots	
**32**	2*α*,3*β*,19*α*-Trihydroxyurs-12-en-23,28-dioic,acid-23-methylester	*A*. *chinensis* Radix [50]	Roots	
**33**	3*β*-Hydroxy-13,28-epoxyurs-11-en-3-ol	*A. kolomikta* [51]	Rhizomes	
**34**	28-Norurs-12-en-3*β*,17*β*-diol	*A. kolomikta* [51]	Rhizomes	
**35**	Triptohypol E	*A. kolomikta* [51]	Rhizomes	
**36**	Neoilexonol	*A. kolomikta* [51]	Rhizomes	
**37**	11*α*-Methoxyurs-12-ene-3*β*,12-diol	*A*. *arguta* [34]	Leaves	
**38**	Ilelatifol A	*A*. *arguta* [34]	Leaves	
**39**	(2*α*,3*β*)-2,3,23-Trihydroxyurs-13(18)-en-28-oic acid	*A. chinensis* Planch [52]	Roots	
**40**	Fupenzic acid	*A*. *chinensis* [25]	Root bark	Antiviral (in vitro) [25]
**41**	Nrsolaldehyde	*A. arguta* [26]	Stems	
**42**	*α*-Amyrin	*A. arguta* [26]	Stems	
**43**	Uvaol	*A. arguta* [26]	Stems	Anti-inflammatory (in vivo) [27] Anticancer (in vitro) [28] Wound healing (in vivo) [29]
**44**	Pseudotaraxasterol	*A. chinensis* Planch [19]	Roots	
**45**	2*α*,3*α*,24-Trihydroxyurs-11-en-13,28-olide	*A. polygama* [23]	Fruit galls	
**46**	2*α*,3*β*-Dihydroxyurs-12-en-28,30-olide	*A. chinensis* [30]	Roots	
**47**	2*α*,3*β*,24-Trihydroxyurs-12-en-28,30-olide	*A. chinensis* [30]	Roots	
**48**	Ehretiolide	*A. kolomikta* [51]	Rhizomes	
**49**	3*β*-Acetoxyurs-11-en-28-oic 13(28)-lactone	*A. kolomikta* [51]	Rhizomes	
**50**	3*β*-Hydroxyurs-12,18-dien-28-oic acid	*A. chinensis* [30]	Roots	
**51**	(2*α*,3*β*,4*α*)-2,3-Dihydroxy-24-norursa-12,18-dien-28-oic acid	*A. valvata* Dunn [47]	Roots	
**52**	2*α*,3*α*,23-Trihydroxy-12,20(30)-ursadien-28-oic acid	*A. polygama* [18] *A. deliciosa* [31]	Fruit galls Peels	Antifungal (in vitro) [31]
**53**	2*α*,3*β*,23-Trihydroxy-12,20(30)-ursadien-28-oic acid	*A. deliciosa* [31]	Peels	Antifungal (in vitro) [31]
**54**	2*α*,3*α*,24-Trihydroxy-12,20(30)-ursadien-28-oic acid	*A. deliciosa* [ 31]	Peels	Antifungal (in vitro) [31]
**55**	2*α*,3*α*,23,24-Tetrahydroxyursa-12,20(30)-dien-28-oic acid	*A. chinensis* Planch [32]	Roots	Anti-tumor (in vitro) [32]
**56**	3*β*-*Trans*-*p*-coumaroyloxy-2*α*,24-dihydroxy-urs-12- en-28-oic acid	*A. polygama* [23]	Fruit galls	
**57**	3-O-*Trans*-*p*-coumaroyl tormentic acid	*A*. *chinensis* Radix [50]	Roots	
**58**	3-O-*Cis-p*-coumaroyl tormentic acid	*A*. *chinensis* Radix [50]	Roots	
**59**	3-O*-Trans-p*-coumaroylasiatic acid	*A. polygama* [18]	Fruit galls	
**60**	23-O-*Trans-p*-coumaroylasiatic acid	*A. arguta* [34]	Leaves	Inhibiting *α*-glucosidase (in vitro) [34]
**61**	Actiniargupene E	*A. arguta* [34]	Leaves	Inhibiting *α*-glucosidase (in vitro) [34]
**62**	Actiniargupene F	*A. arguta* [34]	Leaves	Inhibiting *α*-glucosidase (in vitro) [34]
**63**	Actiniargupene G	*A. arguta* [34]	Leaves	Inhibiting *α*-glucosidase (in vitro) [34]
**64**	3-O-*Trans-p*-coumaroyl actinidic acid	*A. arguta* [13] *A. arguta* [34]	Roots Leaves	
**65**	3-O-*Cis-p*-coumaroylactinidic acid	*A. arguta* [34]	Leaves	Inhibiting *α*-glucosidase (in vitro) [34]
**66**	Actiniargupene A	*A. arguta* [34]	Leaves	Inhibiting *α*-glucosidase (in vitro) [34]
**67**	Actiniargupene B	*A. arguta* [34]	Leaves	Inhibiting *α*-glucosidase (in vitro) [34]
**68**	Actiniargupene C	*A. arguta* [34]	Leaves	Inhibiting *α*-glucosidase (in vitro) [34]
**69**	(+)-Tormentoside	*A. arguta* [53]	Roots	
**70**	(+)-Euscaphic acid-28-O-*β*-d-glucopyranoside	*A. arguta* [53]	Roots	
**71**	2*α*,3*α*,19*α*,24-Tetrahydroxyurs-12-en-28-oic acid 28-O-*β*-d-glucopyranoside	*A. chinensis* Planch [19]	Roots	Inhibiting SKVO3 and TPC-1 cancer cells lines (in vitro) [42]
**72**	2*α*,3*β*,19*α*,23-Tetrahydroxyurs-12-en-28-oic acid 28-O-*β*-d-glucopyranoside	*A*. *chinensis* Radix [50]	Roots	
**73**	2*α*,3*β*,19*α*,23,24-Pentahydroxyurs-12-en-28-oic acid-28-O-*β*-d-glucopyranoside	*A. rufa* [35]	Roots	
**74**	(2*β*,3*α*)-2,3,20,23,24,30-Hexahydroxyurs-12-en-28-oic acid O-*β*-d-glucopyranosyl ester	*A. valvata* Dunn [22]	Roots	Against BEL-7402 and SMMC-7721 tumor cells lines (in vitro) [22]
**75**	2*α*,3*β*,23,30-Tetrahydroxyurs-12,18-diene- 28-oic acid O-*β*-d-glucopyranosyl ester	*A. valvata* Dunn [36]	Roots	Against BEL-7402 and SMMC-7721 tumor cells lines (in vitro) [36]
**76**	30-O-*β*-d-Glucopyranosyloxy-2*α*,3*α*,24- trihydroxyurs-12,18-diene-28-oic acid O-*β*-d-glucopyranosyl ester	*A. valvata* Dunn [36]	Roots	Against BEL-7402 and SMMC-7721 tumor cells lines (in vitro) [36]

Compounds **2**‒**7** are ursane triterpenoids featuring with two hydroxyl groups. Compound **3** (2*α*-Hydroxyursolic acid) was tested for its antiproliferative activity and cytotoxicity in MDA-MB-231 human breast cancer cells through the methylene blue assay. It significantly down-regulated expressions of TRAF2, PCNA, cyclin D1, and CDK4 and up-regulated the expressions of p-ASK1, p-p38, p-p53, and p-21. Furthermore, it induced apoptosis in MDA-MB-231 cell by significantly increasing the Bax/Bcl-2 ratio and inducing the cleaved caspase-3 [12]. Compound **4** exhibited inhibitory activity on pancreatic lipase with an IC_50_ value of 20.42 ± 0.95 μM [13]. It also showed cytotoxicity to human lung adenocarcinoma (A549), ovarian cancer (SK-OV-3), skin melanoma (SK-MEL-2), and colon cancer (HCT-15) cell lines with IC_50_ values ranging from 11.96 to 14.11 μM [14–17].

Compounds **7**‒**14** are trihydroxy-ursolic acid derivatives. In early 1992, Sashida et al. reported the isolation of **8**‒**10**. Compounds **8** and **9** were evaluated for their cytotoxicity against A549 cells, LOVO cells, and HepG2 cells with IC_50_ values of 32.9, 31.6, 35.7 μg/mL respectively for **8** and 34.6, 13.9, 34.5 μg/mL respectively for **9** [18, 19]. Compounds **17**‒**26** are ursolic acids with four or five hydroxy groups. Xu et al. reported **17** from roots of *A. valvata*, this compound exhibited weak cytotoxicity against A549, LOVO and HepG2 cell lines with IC_50_ values of above 100 μg/mL [20]. 2*α*,3*α*,19*α*,24-Tetrahydroxyurs-12-en-28-oic acid **20** was separated from the leaves of *A. valvata* which showed cytotoxicity against PLC, Hep3B, HepG2, HeLa, SW480, MCF-7 and Bel7402 in vitro [21]. A new polyoxygenated triterpenoid (2*β*,3*α*,6*α*)-2,3,6,20,23,30-hexahydroxyurs-12-en-28-oic acid **26** was obtained from the roots of *A. valvata* DUNN, it exhibited moderate cytotoxic activity against BEL-7402 and SMMC-7721 tumor cell lines in vitro [22].

Compound **30** (3*β*-O-acetylursolic acid) was isolated from the fruit galls of *A. polygama* and the structure was elucidated on the basis of chemical and spectral evidence. It was reported to be a mixed-type protein tyrosine phosphatase 1B (PTP1B) inhibitor with an IC_50_ value of 4.8 ± 0.5 *μ*Μ [23, 24]. Isolation of the antiviral active ingredient of *A*. *chinensis* root bark gave fupenzic acid **40**, which showed moderate inactivity under the concentration of 100 μg/mL [25]. Callus tissue from the stems of *A. arguta* (Actinidiaceae) produced three ursane-type triterpenes including ursolaldehyde **41**, *α*-amyrin **42**, and uvaol **43** [26]. Of them, compound **43** showed anti-inflammatory, anticancer, and wound healing activities [27–29]. Anti-inflammatory properties of **43** on DSS-induced colitis and LPS-stimulated macrophages have been explored detailly and completely. It showed excellent potential of NO production inhibition. It could attenuate disease activity index (DAI), colon shortening, colon injury, and colonic myeloperoxidase activity in DSS-induced colitis mice. What’s more, studies on LPS challenged murine macrophage RAW246.7 cells also revealed that uvaol reduces mRNA expression and production of pro-inflammatory cytokines and mediators. These results indicating that uvaol is a prospective anti-inflammatory agent for colonic inflammation [27]. Guided by the hepatoprotective activity, the phytochemical study on the roots of *A. chinensis* led to the isolation of two new compounds 2*α*,3*β*-dihydroxyurs-12-en-28,30-olide **46**, 2*α*,3*β*,24-trihydroxyurs-12-en-28,30-olide **47** and 3*β*-hydroxyurs-12,18-dien-28-oic acid **50** [30]. Compounds **52**‒**54** showed antifungal activity against *C. musae* at 3 μg/mL [31]. A new compound 2*α*,3*α*,23,24 -tetrahydroxyursa-12,20(30)-dien-28-oic acid **55** was isolated from the roots of *A. chinensis* Planch. It exhibited moderate antitumor activities against five human cancer cell lines (HepG2, A549, MCF-7, SK-OV-3, and HeLa) with IC_50_ values of 19.62 ± 0.81, 18.86 ± 1.56, 45.94 ± 3.62, 62.41 ± 2.29, and 28.74 ± 1.07 μM, respectively [32].

Compounds **59**‒**63** are actinidic acid derivatives with a phenylpropanoid unit that were identified as 3-O-*trans-p*-coumaroylasiatic acid **59**, 23-O-*trans-p*-coumaroylasiatic acid **60**, actiniargupene E **61**, actiniargupene F **62**, and actiniargupene G **63** from the leaves of *A. arguta*. All the compounds showed inhibitory effects on *α*-glucosidase activity. Among them compound **59** showed most potentially inhibitory activity on *α*-glucosidase with an IC_50_ of 81.3 ± 2.7 μM, equal to that of the positive control (acarbose, 72.8 ± 3.1 μM) [33]. The structure–activity relationship suggested that triterpenoids with a phenylpropanoid moiety exhibited more potent effects than those without such a unit [34]. Compound **71** showed potent cytotoxic activity against human SKVO3 and TPC-1 cancer cell lines with IC_50_ values of 10.99 and 14.34 μM, respectively [19, 35]. Compound **74** exhibited moderate cytotoxic activity against BEL-7402 and SMMC-7721 tumor cell lines [22]. Compounds **75** and **76** were isolated from roots of *A. valvata* Dunn. They exhibited moderate cytotoxic activity in vitro against BEL-7402 and SMMC-7721 tumor cell line [36].

#### Oleanane Triterpenoids

**Fig. 3:**
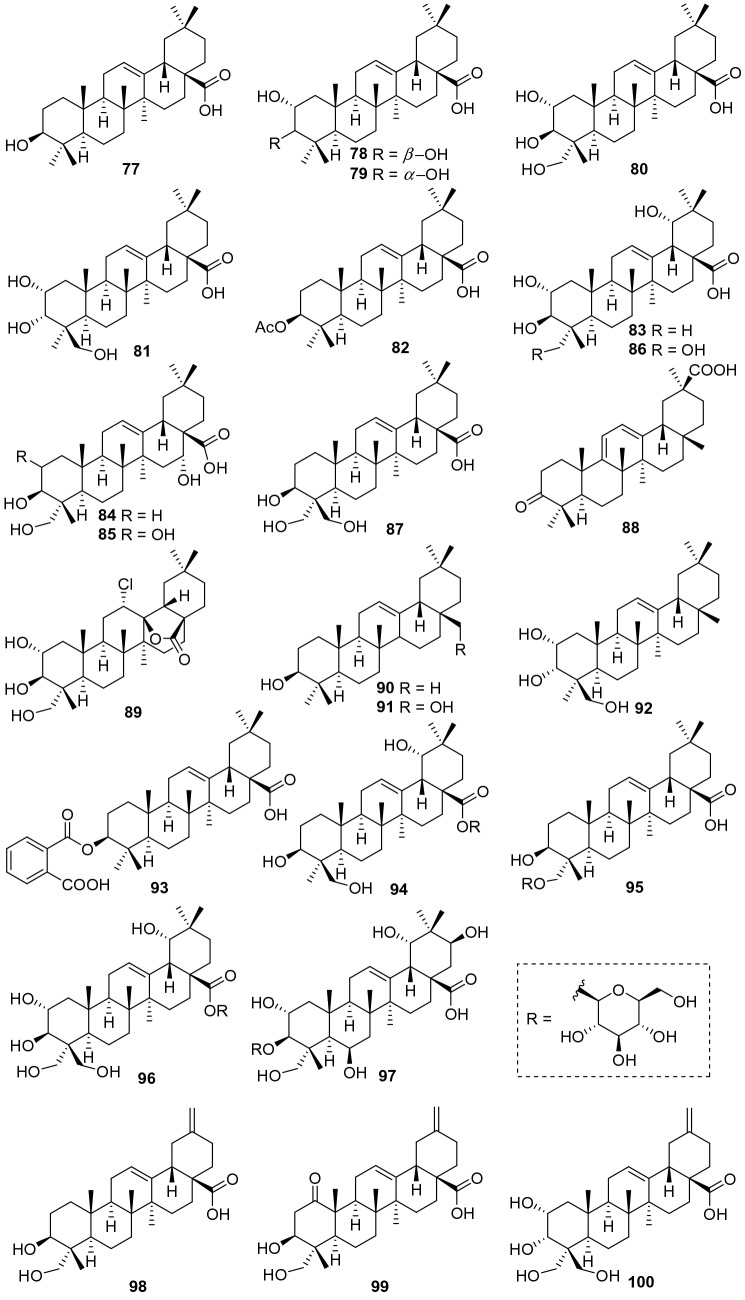
Structures of oleanane triterpenoids **77**‒**100** from *Actinidia* plants

Oleanane-type triterpenoids also possessed a 6/6/6/6/6 pentacyclic carbon skeleton. Unlike ursane triterpenes, oleanane-type triterpenoids have two methyl groups at the C-20 position instead of each one at the C-19 and C-20, respectively. So far, a total of 24 oleanane-type triterpenoids have been identified from *Actinidia* plants (**77**‒**100**, Fig. 3, Table 2). The most representative compound is oleanolic acid **77**. It was found from callus tissue from the stems of *A. arguta*, together with 2*α*,3*β*-dihydroxyolean-12-en-28-oic acid **78** [26]. Oleanolic acid **77** is abundant in nature and exhibits a wide range of biological activities including anti-inflammatory [54], anti-hypertension [55], anti-tumor [56, 57], neuroprotection [58], and anti-cholesterol activities [59]. Oleanolic acid was performed to test the effect on apoptosis and autophagy of SMMC-7721 Hepatoma cells. It can significantly inhibit the growth of liver cancer SMMC-7721 cells and induce autophagy and apoptosis [57]. Compound **78** also showed anti-tumor and anti-inflammatory activities [60, 61]. Lim et al. have demonstrated that **78** showed very strong anti-tumor-promoting activity with an IC_50_ of 0.1 mg/mL [60].

**Table 2:** Information of oleanane triterpenoids from *Actinidia* plants

No.	Compound name	Species Refs.	Part	Bioactivity Refs.
**77**	Oleanolic acid	*A. arguta* [26]	Stems	Anti-inflammatory (in vitro) [54] Anti-hypertension (in vivo) [55] Anti-tumor (in vitro) [56, 57] Neuroprotection(in vitro) [58] Anti-cholesterol(in vitro) [59]
**78**	2*α*,3*β*-Dihydroxyolean-12-en-28-oic acid	*A. arguta* [26]	Stems	
**79**	2*α*,3*α*-Dihydroxyolean-12-en-28-oic acid	*A. chinensis* Planch [63]	Fruits	
**80**	2*α*,3*β*,23-Trihydroxyolean-12-en-28-oic acid	*A. deliciosa* [31]	Peels	Antifungal (in vitro) [31]
**81**	2*α*,3*α*,24-Trihydroxyolean-12-en-28-oic acid	*A. deliciosa* [31]	Peels	Antifungal (in vitro) [31]
**82**	3*β*-O-Acetyloleanolic acid	*A. arguta* [64] *A. chinensis* [45]	Stems Roots	Anti-angiogenesis (in vitro) [45]
**83**	2*α*,3*β*,19-Trihydroxyolean-12-en-28-oic acid	*A. chinensis* [45]	Roots	Anti-angiogenesis (in vitro) [45]
**84**	(3*β*,4*α*,16*α*)-3,16,23-Trihydroxyolean-12- en-28-oic acid	*A. valvata* Dunn [47]	Roots	
**85**	(2*β*, 3*β*, 4*α*, 16*α*)-2, 3, 16, 23-Tetrahydroxyolean-12-en-28-oic acid	*A. valvata* Dunn [47]	Roots	
**86**	2*α*,3*β*,19*α*,23-Tetrahydroxyoleanolic acid	*A. deliciosa* [65]	Roots	
**87**	3*β*,23,24-Trihydroxyl-12-oleanen-28-oic acid	*A. eriantha* Benth [62]	Roots	Anti-angiogenesis (in vitro) [62]
**88**	9(11),12-Diene-30-oic acid	*A*. *chinensis* [25]	Roots bark	Anti-viral (in vitro) [25]
**89**	12*α*-Chloro-2*α*,3*β*,13*β*,23-tetrahydroxyolean-28-oic acid-13-lactone	*A. chinensis* Planch [19]	Roots	Anti-catalysis (in vitro) [43]
**90**	*β*-Amyrin	*A. arguta* [26]	Stems	
**91**	Erythrodiol	*A. kolomikta* [51]	Dried rhizomes	
**92**	12-Oleanene 2*α*,3*α*,24-triol	*A. macrosperma* [66]	Roots	
**93**	3*β*-(2-Carboxybenzoyloxy) oleanolic acid	*A. chinensis* [25]	Root bark	Anti-phytoviral (in vitro) [25]
**94**	Spathodic acid-28-O-*β*-d-glucopyranoside	*A. chinensis* [25]	Root bark	Anti-phytoviral (in vitro) [25]
**95**	Oleanolic acid-23-O-*β*-d-glucopyranoside	*A. eriantha* Benth [62]	Roots	Anti-angiogenesis (in vitro) [62]
**96**	2*α*,3*β*,19*α*,23,24-Pentahydroxy-12-oleanen 28-oic acid 28-*β*-d-glucopyranosyl	*A*. *chinensis* Radix [50]	Roots	
**97**	3-O-*β*-d-Glucopyranosyl-2*α*,3*β*,6*β*,19*α*,21*β*,23-hexahydroxylolean-12-en-28-oic acid	*A*. *kolomikta* [67]	Leaves	
**98**	3*β*,23-Dihydroxy-30-norolean-12,20(29)- dien-28-oic acid	*A*. *chinensis* Radix [50]	Roots	
**99**	3*β*,23-Dihydroxy-1-oxo-30-norolean- 12,20(29)-dien-28-oic acid	*A*. *chinensis* Radix [50]	Roots	
**100**	2*α*,3*α*,23,24-Tetrahydroxy-30-norolean- 12,20(29)-dien-28-oic acid	*A*. *chinensis* Radix [50]	Roots	

Bioassay- and ^1^H NMR-guided fractionation of the methanol extract afforded two oleanolic acids of 2*α*,3*β*,23-trihydroxyolean-12-en-28-oic acid **80** and 2*α*,3*α*,24-trihydroxyolean-12-en-28-oic acid **81**, showing antifungal activity against *C. musae* at 3 μg/mL [31]. The EtOAc extract of the roots of *A. eriantha* Benth exhibited potent growth inhibitory activity against SGC7901 cells, CNE2 cells and HUVECs cells. From which, compound **87** (3*β*,23,24-trihydroxyl-12-oleanen-28-oic acid) was identified [62]. Compound **88** was extracted from the roots bark of *A. chinensis*, which showed anti-viral activity [25]. A new triterpenoid 12*α*-chloro-2*α*,3*β*,13*β*,23 -tetrahydroxyolean-28-oic acid-13-lactone **89** was extracted from the roots of *A. chinensis* Planch (Actinidiaceae). It was tested for cytochrome P450 (CYPs) enzyme inhibitory activity in later years, which could significantly inhibit the catalytic activities of CYP3A4 to < 10% of its control activities [19, 52].

3*β*-(2-Carboxybenzoyloxy) oleanolic acid **93** and spathodic acid-28-O-*β*-d-glucopyranoside **94** were extracted from the root bark of *A. chinensis*. The anti-phytoviral activity test indicated that **94** showed potent activity on TMV, and CMV with inactivation effect of 46.67 ± 1.05, and 45.79 ± 2.23 (100 mg/L), compared to ningnanmycin with inactivation effect of 30.15 ± 1.16 and 27.18 ± 1.02 (100 mg/L) respectively [25]. 3*β*,23-Dihydroxy- -30-norolean-12,20(29)-dien-28-oic acid **98**, 3*β*,23-dihydroxy-1-oxo-30-norolean-12,20(29)-dien-28-oic acid **99**, and 2*α*,3*α*,23,24-tetrahydroxy-30-norolean-12,20(29)-dien-28-oicacid **100** are three one-carbon-degraded oleanane triterpenoids that were identified from *A. chinensis* Radix for the first time [50].

#### Lupane Triterpenoids

**Fig. 4:**
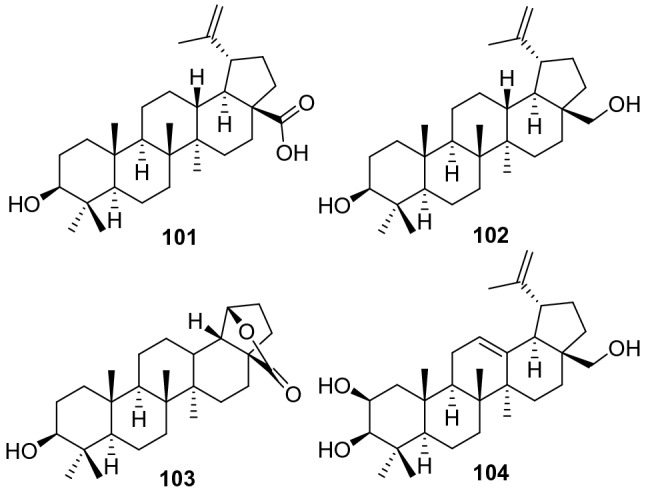
Structures of lupane triterpenoids **101**‒**104** from *Actinidia* plants

Lupane triterpenoids possess a 6/6/6/6/5-fused carbon skeleton. Compared with ursane and oleanane triterpenoids, the number of lupane triterpenoids in the *Actinidia* plants is much smaller, only four related compounds have been identified (**101**‒**104**, Fig. 4, Table 3). Three of them (**101**‒**103**) were identified from the rhizomes of *A. kolomikta* [51]. Betulinic acid **101** is one of the most representative compound of lupane triterpenes, it has been extensively studied in recent years based on the wide biological activities including anti-inflammatory, antitumor, anti-HIV, anti-diabetic and antimalarial activities [68–73]. Much attention as a molecular target about protein tyrosine phosphatase 1B had been paid to the treatment of insulin resistance diseases because of its critical roles in negatively regulating insulin- and leptin-signaling cascades. Betulinic acid showed significant PTP1B inhibitory activity, with IC_50_ values of 3.5 μM [24].

**Table 3:** Information of lupane triterpenoids from *Actinidia* Plants

No.	Compound name	Species Refs.	Part	Bioactivity Refs.
**101**	Betulinic acid	*A. kolomikta* [51]	Rhizomes	Anti-inflammatory (in vitro) [68] Antitumor(in vitro) [69] Anti-HIV(in vitro) [72] Anti-diabetic(in vivo) [73]
**102**	Betulin	*A. kolomikta* [51]	Rhizomes	Anti-tumor(in vitro) [74–76]
**103**	Diospyrolide	*A. kolomikta* [51]	Rhizomes	
**104**	Lupa-12,20(30)-diene-2*β*,3*β*,28-triol	*A. deliciosa* [48]	Roots	

#### Other Terpenoids

**Fig. 5:**
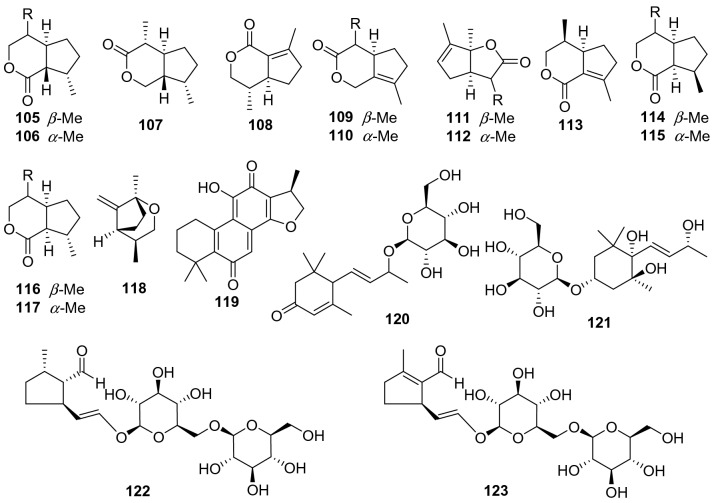
Structures of other terpenoids **105**‒**123** from *Actinidia* plants

A total of 19 other terpenoids including iridoids, diterpenoids, and their glycosides have been found from *Actinidia* plants (**105 − 123**, Fig. 5, Table 4). None of these compounds have good biological activities, only compound **120** showed certain anti-angiogenesis activity [77].

**Table 4:** Information of other triterpenoids from *Actinidia* plants

No.	Compound name	Species Refs.	Part	Bioactivity Refs.
**105**	Dihydroepinepetalactone	*A. polygama* [78]	Fresh fruits	
**106**	Isodihydroepinepetalactone	*A. polygama* [78]	Fresh fruits	
**107**	Isoepiiridomyrmecin	*A. polygama* [78]	Fresh fruits	
**108**	Isoneonepetalactone	*A. polygama* [78]	Fresh fruits	
**109**	Dehydroiridomyrmecin	*A. polygama* [78]	Fresh fruits	
**110**	Isodehydroiridomyrmecin	*A. polygama* [78]	Fresh fruits	
**111**	Actinidialactone	*A. polygama* [78]	Fresh fruits	
**112**	Isoactinidialactone	*A. polygama* [78]	Fresh fruits	
**113**	Neonepetalactone	*A. polygama* [78]	Fresh fruits	
**114**	Dihydronepetalactone	*A. polygama* [78]	Fresh fruits	
**115**	Isodihydronepetalactone	*A. polygama* [78]	Fresh fruits	
**116**	Iridomyrmecin	*A. polygama* [78]	Fresh fruits	
**117**	Isoiridomyrmecin	*A. polygama* [78]	Fresh fruits	
**118**	Matatabiether	*A. polygama* [79]	Leaves and galls	
**119**	(*R*)-1,2,6,7,8,9-Hexahydro-10-hydroxy-1,6,6-trimethylphenanthro[1,2-b]furan-5,11-dione	*A. valvata*Dunn [47]	Roots	
**120**	(6*R*,7*E*,9*S*)-6,9-Hydroxy-megastigman-4,7-dien-3-one-9-O-*β*-glucopyranoside	*A. eriantha* Benth [62]	Roots	Anti-angiogenesis (in vitro) [62]
**121**	Kiwiionoside	*A. chinensis* [80]	Fresh leaves	
**122**	Iridodialo-*β*-d-gentiobioside	*A. polygama* [77]	Leaves	
**123**	Dehydroiridodialo-*β*-d-gentiobioside	*A. polygama* [77]	Leaves	

### Steroids

**Fig. 6:**
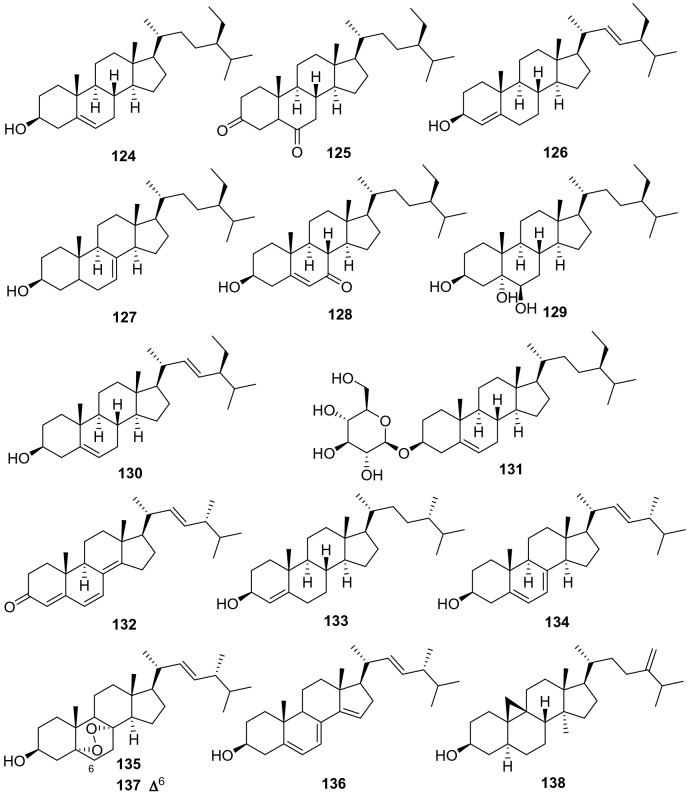
Structures of steroids **124**‒**138** from *Actinidia* plants

*β*-Sitosterol **124** is a very normal phytosterol almost distributed in all plants. Eight phytosterols have been obtained from the *Actinidia* plants (**124**‒**131**, Fig. 6, Table 5). Pharmacological studies on these steroids have demonstrated that *β*-sitosterol showed various bioactivities including anti-inflammatory, anti-cancer, antimicrobial and anti-diabetic properties [81–88]. A study suggested that *β*-sitosterol may serve as a potential therapeutic in the treatment of acute organ damages [82].

**Table 5:** Information of steroids from *Actinidia* plants

No.	Compound name	Species Refs.	Part	Bioactivity Refs.
**124**	*β*-Sitosterol	*A. eriantha* [7]	Roots	Anti-inflammatory (In vivo) [84] Anticancer (in vitro) [85] Anti-microbial (in vitro) [86] Anti-diabetic (In vivo) [88]
**125**	Stigmast-3,6-dione	*A. chrysantha* [42]	Roots	
**126**	Masterol	*A. deliciosa* [89]	Peels	
**127**	Stigmast-7-en-3*β*-ol	*A. deliciosa* [89]	Peels	
**128**	3*β*-Hydroxystigmast-5-en-7-one	*A. chinensis* Planch [52]	Roots	
**129**	Sitostanetriol	*A*. *kolomikta* [51]	Dried rhizomes	
**130**	(24*R*)-Stigmast-4-en-3-one	*A*. *kolomikta* [51]	Dried rhizomes	
**131**	Stigmasterol	*A. arguta* [26]	Stems	Anti-inflammatory (in vitro) [90]
**132**	Daucosterol	*A. eriantha* [40] *A. chinensis* [44]	Roots Roots	
**133**	Campesterol	*A. deliciosa* [89]	Peels	
**134**	Ergosterol	*A. deliciosa* [89]	Peels	
**135**	Ergost-22-en-3-ol	*A. deliciosa* [89]	Peels	
**136**	5,7,14,22-Ergostatetraen-3*β*-ol	*A. deliciosa* [89]	Peels	
**137**	5,8-Epidioxyergosta-6,22-dien-3*β*-ol	*A*. *kolomikta* [51]	Dried rhizomes	
**138**	24-Methylene-pollinastanol	*A*. *kolomikta* [51]	Dried rhizomes	

In addition to phytosterols, seven normal ergosterols (**132**‒**137**, Fig. 6, Table 5) were obtained from peel or rhizomes of kiwifruit plants. It is well known that ergosterols should be fungal products. Compounds **132**‒**137** may be produced by fungal infected kiwifruit plants.

### Phenols

#### Catechins and Epicatechins

**Fig. 7:**
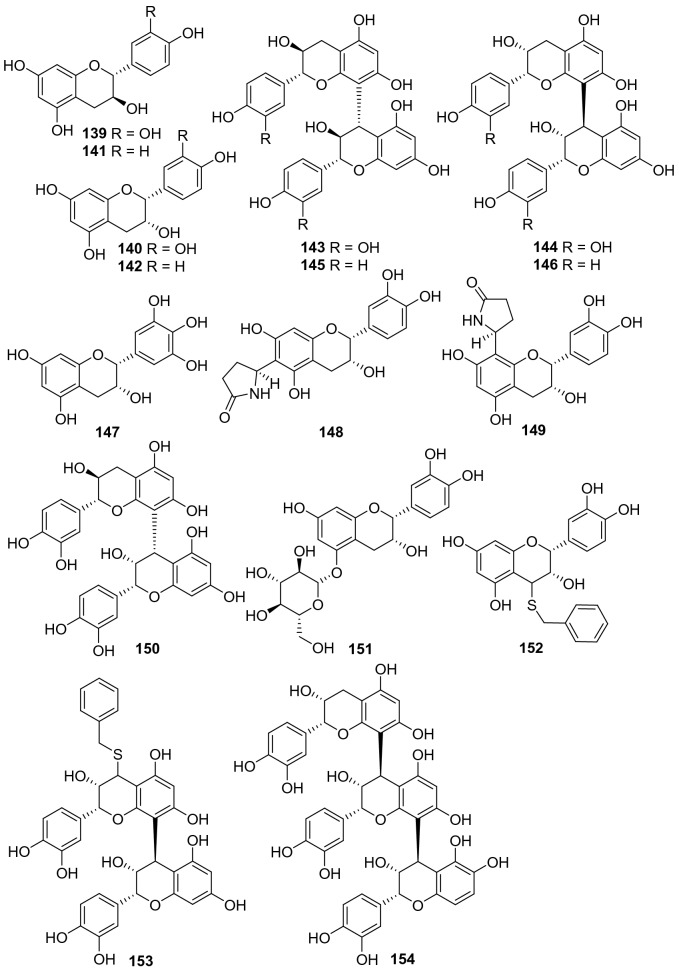
Structures of catechins and epicatechins **139**‒**154** from *Actinidia* plants

A total of 16 related compounds (**139**‒**154**) have been obtained from kiwifruit plants (Fig. 7, Table 6). Compounds **148** and **149** possessed a novel structure featuring with a pyrrolidin-2-one substituent at C-6 and C-8, respectively. Compounds **152** and **153** were two sulfur-containing catechins that was rare in nature. Pharmacological studies have revealed that (+)-catechin **139** and (−)-epi-catechin **140** showed nitric oxide inhibitory activity in LPS stimulated RAW 264.7 cell with IC_50_ values of 26.61 and 25.30 μg/mL, respectively [53, 91]. Compound **147** showed moderate radical scavenging and antioxidant capabilities by measuring their capacity to scavenge DPPH and anion superoxide radical and to reduce a Mo(VI) salt [89]. Two new flavan-3-ols, 6-(2-pyrrolidinone-5-yl)-(−)-epicatechin **148** and 8-(2-pyrrolidinone-5-yl)-(−)-epicatechin **149**, as well as proanthocyanidin B-4 **150**, were isolated from an EtOAc-soluble extract of the roots of *A. arguta*. The isolates were tested in vitro for their inhibitory activity on the formation of advanced glycation end products (AGEs). All of them exhibited significant inhibitory activity against AGEs formation with IC_50_ values ranging from 10.1 to 125.2 μM [92].

**Table 6:** Information of catechins and epicatechins from *Actinidia* plants

No.	Compound name	Species Refs.	Part	Bioactivity Refs.
**139**	(+)-Catechin	*A. arguta* [41]	Roots	Anti-DPPH radical and inhibiting nitric oxide (in vitro) [57] Anti-bacteria (in vitro) [93]
**140**	(−)-Epi-catechin	*A. arguta* [41]	Roots	Anti-DPPH radical and inhibiting nitric oxide (in vitro) [57]
**141**	(+)-Afzelechin	*A. chinensis* Planch [94]	Roots	
**142**	(−)-*Epi*-afzelechin	*A. chinensis* Planch [94]	Roots	
**143**	(+)-Catechin(4*α* → 8)(+)-catechin	*A. chinensis* Planch [94]	Roots	
**144**	(−)-*Epi*-catechin(4*β* → 8)(−)-epi-catechin	*A. chinensis* Planch [94]	Roots	
**145**	(+)-Afzelechin(4*α* → 8) (+)-afzelechin	*A. chinensis* Planch [94]	Roots	
**146**	(−)-*Epi*-afzelechin(4*β* → 8)(−)-epi-afzelechin	*A. chinensis* Planch [94]	Roots	
**147**	Gallocatechin	*A. deliciosa* [89]	Peels	Radical scavenging and antioxidant (in vitro) [89]
**148**	6-(2-Pyrrolidinone-5-yl)-(−)-epicatechin	*A. arguta* [92]	Roots	Against advanced glycation-end (in vitro) [92]
**149**	8-(2-Pyrrolidinone-5-yl)-(−)-epicatechin	*A. arguta* [92]	Roots	Against advanced glycation-end (in vitro) [92]
**150**	Proanthocyanidin B-4	*A. arguta* [92]	Roots	Against advanced glycation-end (in vitro) [92]
**151**	(−)-*Epi*-Catechin-5-O-*β*-D-glucopyranoside	*A. arguta* [92]	Roots	Against advanced glycation-end (in vitro) [92]
**152**	Benzylthio-(−)-epicatechol	*A. chinensis* [95]	Vegetative parts	
**153**	4′-Benzylthioprocyanidol B2	*A. chinensis* [95]	Vegetative parts	
**154**	Procyanidol C1	*A. chinensis* [95]	Vegetative parts	

#### Flavones, Isoflavones, and Flavonols

**Fig. 8:**
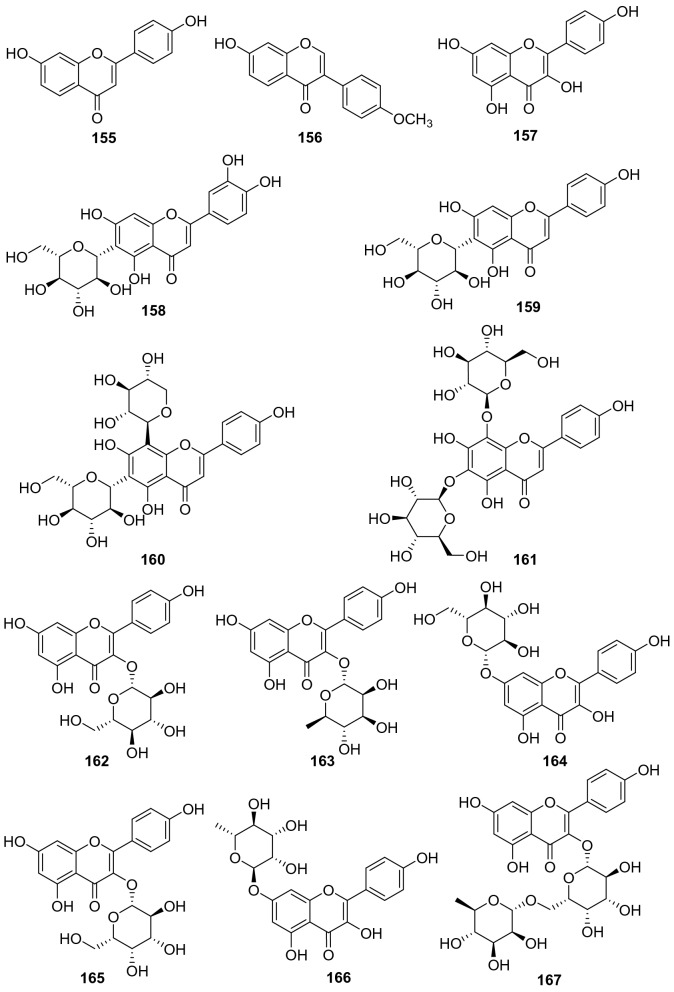

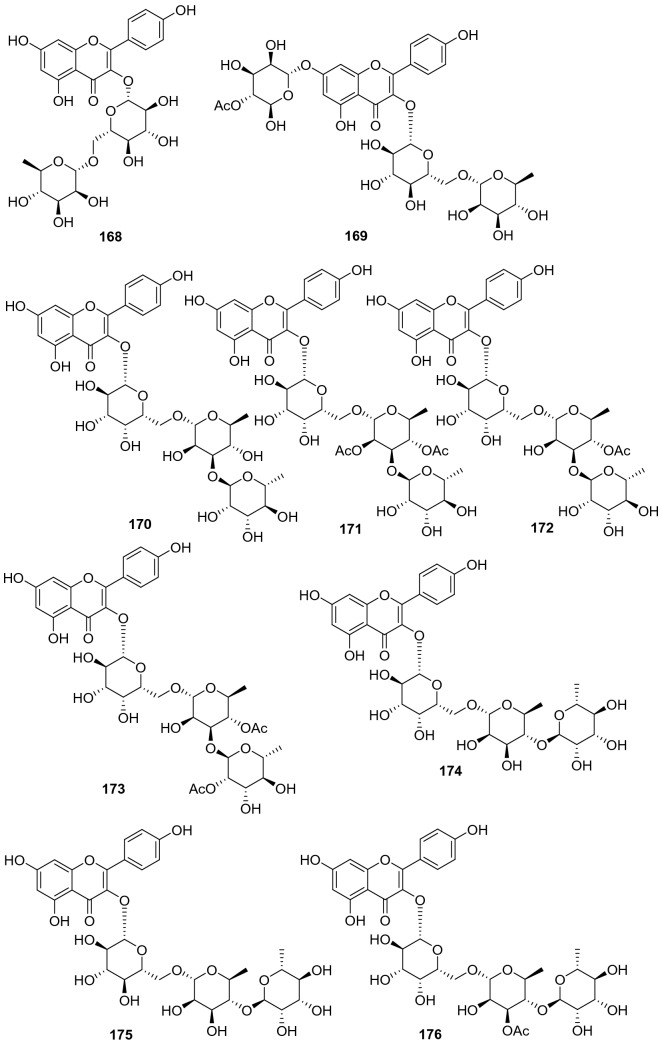

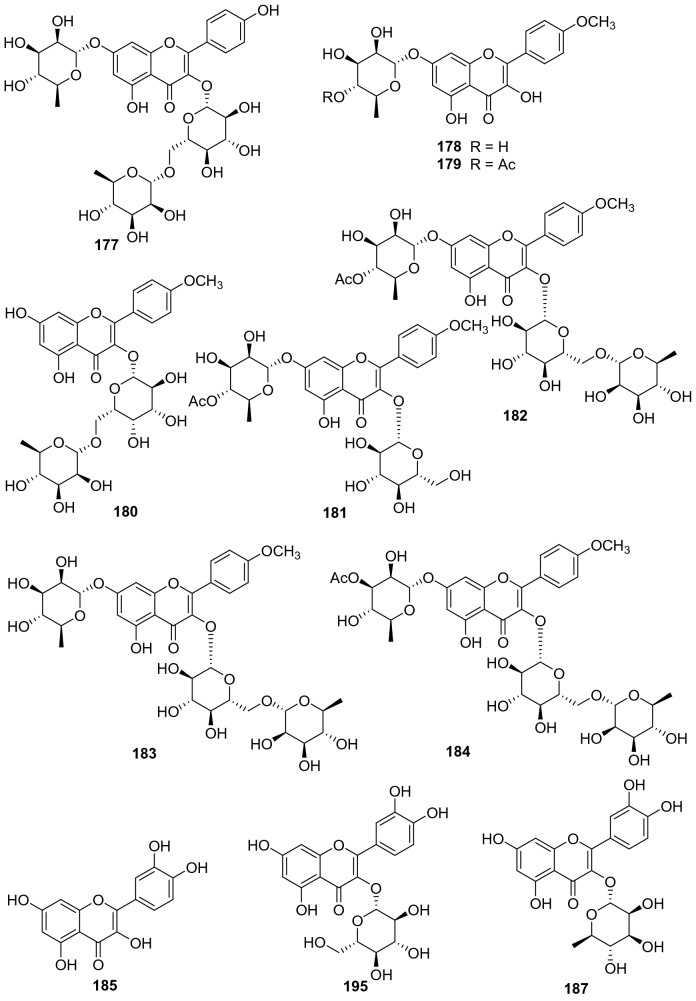

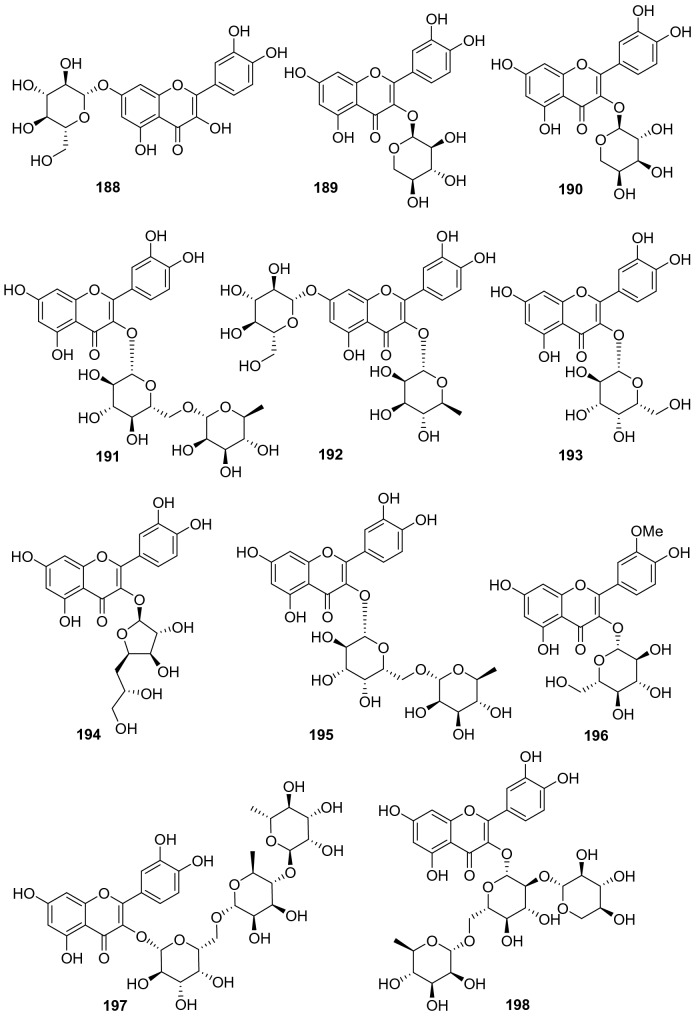

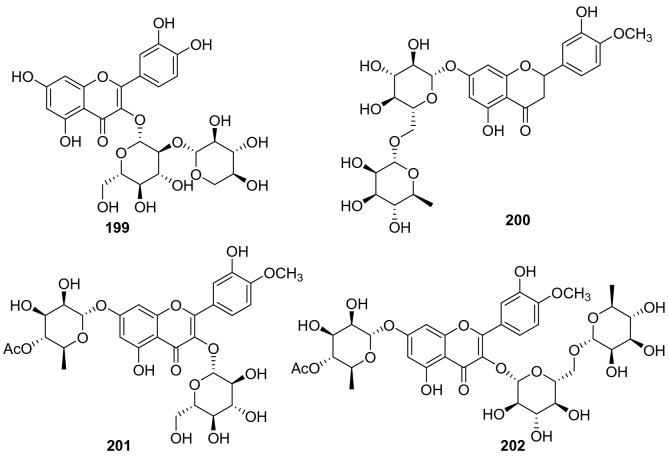
Structures of flavones, isoflavones, and flavonols **155**‒**202** from *Actinidia* plants

A total of 48 flavone derivatives have been identified from kiwifruit plants, most of which are glycosides (**155**‒**202**, Fig. 8, Table 7). Pharmacological studies indicated that these compounds, particularly kaempferol and its derivative, had a wide range of biological activities including antiproliferation, antioxidation, anti-inflammation, anticancer, anti-free radical, and neuroprotection activities [96–99]. Kaempferol **157** was found to prevent neurotoxicity by several ways which was able to completely block *N*-methyl-D-aspartate (NMDA)-induced neuronal toxicity and potently inhibited MAO (monoamine oxidase) with the IC_50_ of 0.8 μM [99]. Two novel flavonoids **171** and **172** were separated from the leaves of *A. valvata* Dunn. They exhibited dose-dependent activity in scavenging 1,1-diphenyl-2-picrylhydrazyl (DPPH) free radicals, superoxide anion radicals, and hydroxyl radicals, and inhibited lipid peroxidation of mouse liver homogenate in vitro [100]. Compounds **178** [101] and **179** [102] were two new compounds obtained from the leaves of *A. kolomikta*. The latter was screened for its protective effect on human erythrocytes against AAPH-induced hemolysis, which could slow the hemolysis induced by AAPH [102]. Eerduna et al. evaluated the effects of compound **182** on acute myocardial infarction in rats, the groups treated with **182** showed a dose-dependent reduction in myocardial infarct size model, markedly inhibited the elevation of the activity of creatine kinase, troponin T level, and the content of malondialdehyde induced by AMI [103]. Compound **182** also showed a capacity to increase the activities of superoxide dismutase, catalase, and endothelial nitric oxide synthase [104]. Lim et al. tested the DPPH radical scavenging activity and nitric oxide production inhibitory activity in IFN-*γ*, LPS stimulated RAW 264.7 cell of quercetin **185**, quercetin-3-O-*β*-d-glucoside **186**, and quercetin 3-O-*β*-d-galactoside **193** with IC_50_ value of 20.41, 18.23, and 30.46 μg/mL, respectively [91].

**Table 7:** Information of flavones, isoflavones, and flavonols from *Actinidia* plants

No.	Compound name	Species Refs.	Part	Bioactivity Refs.
**155**	7,4′-Dihydroxyflavone	*A. chinensis* Planch [105]	Roots	Anti-inflammatory (in vitro) [106]
**156**	Formononetin	*A. chinensis* Planch [105]	Roots	
**157**	Kaempferol	*A*. *deliciosa* [107]	Leaves	Antiproliferation human tumor cell (in vitro) [96] Antioxidant and anti-inflammatory (in vitro) [97] Anticancer (in vitro) [98] Neuroprotection (in vitro) [99]
**158**	6-C-Glucose-5,7,3′,4′-tetrahydroxy flavone	*A. arguta* [108]	Roots	
**159**	6-C-Glucose-5,7,4′-trihydroxy flavone	*A. arguta* [108]	Roots	
**160**	6-C-Glycopyranosyl-8-C-xyloeyl apigenin	*A. polygama* [109]	Leaves	
**161**	Vicenin-I	*A. polygama* [110]	Aerial Parts	
**162**	Kaempferol 3-O-glucoside	*A*. *deliciosa* [107]	Leaves	
**163**	Kaempferol 3-O-rhamnoside	*A*. *deliciosa* [107]	Leaves	
**164**	Kaempferol 7-O-glucoside	*A*. *deliciosa* [107]	Leaves	
**165**	Kaempferol 3-O-*β*-d-galactopyranoside	*A. polygama* [109]	Leaves	
**166**	Kaempferol-7-O-*β*-l-rhamnoside	*A. kolomikta* [102]	Leaves	
**167**	Kaempnferol-3-O-*α*-l-rhamnopyranosyl-(1 → 6)-*β*-d-galactopyranoside	*A. polygama* [109]	Leaves	
**168**	Kaempferol 3-O-rutinoside	*A*. *deliciosa* [107]	Leaves	
**169**	Kaempferol-7-O-(4″-O-acetylrhamnosyl)-3-O-rutinoside	*A. kolomikta* [111]	Leaves	
**170**	Kaempferol-3-O-*α*-l-rhamnopyranosyl- (1 → 3)-*α*-l-rhamnopyranosyl- (1 → 6)-*β*-d-galactopyranoside	*A. polygama* [109]	Leaves	
**171**	Kaempferol 3-O-*α*-l-rhamnopyranosyl-(1 → 3) (2,4-di-O-acetyl-*α*-l-rhamnopyranosyl) (1 → 6) *β*-d-galactopyranoside	*A. valvata* Dunn [100]	Leaves	Anti-free radical (in vitro) [100]
**172**	Kaempferol 3-O-*α*-l-rhamnopyranosyl (1 → 3) (4-O-acetyl-*α*-l-rhamnopyranosyl) (1 → 6) *β*-D-galactopyranoside	*A. valvata* Dunn [100]	Leaves	Anti-free radical (in vitro) [100]
**173**	Kaempferol 3-O-[*α*-l-rhamnopyranosyl-(1 → 3)- (4-O-acetyl)-O-*α*-l-rhamnopyranosyl-(1 → 6)- O-acetyl)-O-*β*-d-galactopyranoside]	*A. polygama* [110]	Aerial parts	
**174**	Kaempferol 3-O-[*α*-rhamnopyranosyl-(1 → 4)- rhamnopyranosyl-(1 → 6)-*β*-galactopyranoside]	*A. deliciosa* [112]	Leaves	
**175**	Kaempferol 3-O-[*α*-rhamnopyranosyl-(1 → 4)- rhamnopyranosyl-(1 → 6)-*β*-glucopyranoside]	*A. deliciosa* [112]	Leaves	
**176**	Kaempferol 3-O-[*α*-rhamnopyranosyl-(1 → 4)-3‴ -O-acetyl-*α*-rhamnopyranosyl-(1 → 6)-*β*-galactop-yranoside]	*A. deliciosa* [112]	Leaves	
**177**	Kaempferol-7-O-*α*-l-rhamnosyl-3-O-rutinoside	*A. kolomikta* [102]	Leaves	
**178**	Kaempferide-7-O-rhamnoside	*A. kolomikta* [101]	Leaves	
**179**	Kaempferide-7-O-(4″-O-acetyl)-*α*-l-rhamnoside	*A. kolomikta* [102]	Leaves	Against AAPH-​induced hemolysis (in vitro) [102]
**180**	Kaempferide-3-O-rutinoside	*A. kolomikta* [113]	Leaves	Anti-free radical (in vitro) [113]
**181**	Kaempferide-7-O-(4″-O-acetyl-rhamnosyl)-3-O-glucoside	*A. kolomikta* [113]	Leaves	Anti-free radical (in vitro) [113]
**182**	Kaempferide-7-O-(4″-O-acetyl-rhamnosyl)-3-O-rutinoside	*A. kolomikta* [113]	Leaves	Anti-free radical (in vitro) [113] Reducing myocardial infarction (in vivo) [104]
**183**	Kaempferide-7-O-rhamnosyl-3-O-rutinoside	*A. kolomikta* [101]	Leaves	
**184**	Kaempferide-7-O-(3″-O-acetylrhamnosyl)-3-O-rutinoside	*A. kolomikta* [111]	Leaves	
**185**	Quercetin	*A. deliciosa* [107]	Leaves	
**186**	Quercetin-3-O-*β*-d-glucoside	*A. deliciosa* [107]	Leaves	
**187**	Quercetin 3-O-rhamnoside	*A. deliciosa* [107]	Leaves	
**188**	Quercetin 7-O-glucoside	*A. deliciosa* [107]	Leaves	
**189**	Quercetin 3-O-xyloside	*A. deliciosa* [107]	Leaves	
**190**	Quercetin 3-O-arabinoside	*A. deliciosa* [107]	Leaves	
**191**	Quercetin 3-O-rutinoside	*A. deliciosa* [107]	Leaves	
**192**	Quercetin 3-O-rhamnoside 7-O-glucoside	*A. deliciosa* [107]	Leaves	
**193**	Quercetin 3-O-*β*-d-galactoside	*A. polygama* [110]	Aerial parts	Anti-DPPH radical and nitric oxide production (in vitro) [110]
**194**	Quercetin 3-O-*β*-d-glucofuranoside (isoquercitrin)	*A. chinensis* Planch [114]		
**195**	Quercetin 3-O-*α*-l-rhamnopyranosyl-(1 → 6)-*β*-d-galactopyranoside	*A. arguta* [108]	Roots	
**196**	Isorhamnetin-3-O-*β*-d-glucoside	*A. kolomikta* [102]	Leaves	
**197**	Quercetin3-O-[*α*-rhamnopyranosyl-(1 → 4) -rhamnopyranosyl-(1 → 6)-*β*-galactopyranoside	*A. deliciosa* [112]	Leaves	
**198**	Quercetin 3-O-*β*-d-[2^G^-O-*β*-d-xylopyranosyl-6^G^-O-*α*-l-rhamnopyranosyl] glucopyranoside	*A. arguta* [115]	Leaves	
**199**	Quercetin 3-O-*β*-d-xylopyranosyl- (1 → 2)-O-*β*-d-glucopyranoside	*A. arguta* [115]	Leaves	
**200**	7-O-[*β*-d-Pyranrhamnose-(1 → 6)-*β*-D-pyran glucose]-5,3′-dihydroxy-4′-methoxy two dihydrogen flavone	*A. arguta* [108]	Roots	
**201**	4′-Methoxyl-quercetin-7-(4″-O-acetylrhamnosyl)-3-O-*β*-D-glucopyranoside	*A. kolomikta* [111]	Leaves	
**202**	4′-Methoxyl-quercetin-7-(4″-O-acetylrhamnosyl)-3-O-rutinoside	*A. kolomikta* [111]	Leaves	

#### Xanthones

**Fig. 9:**
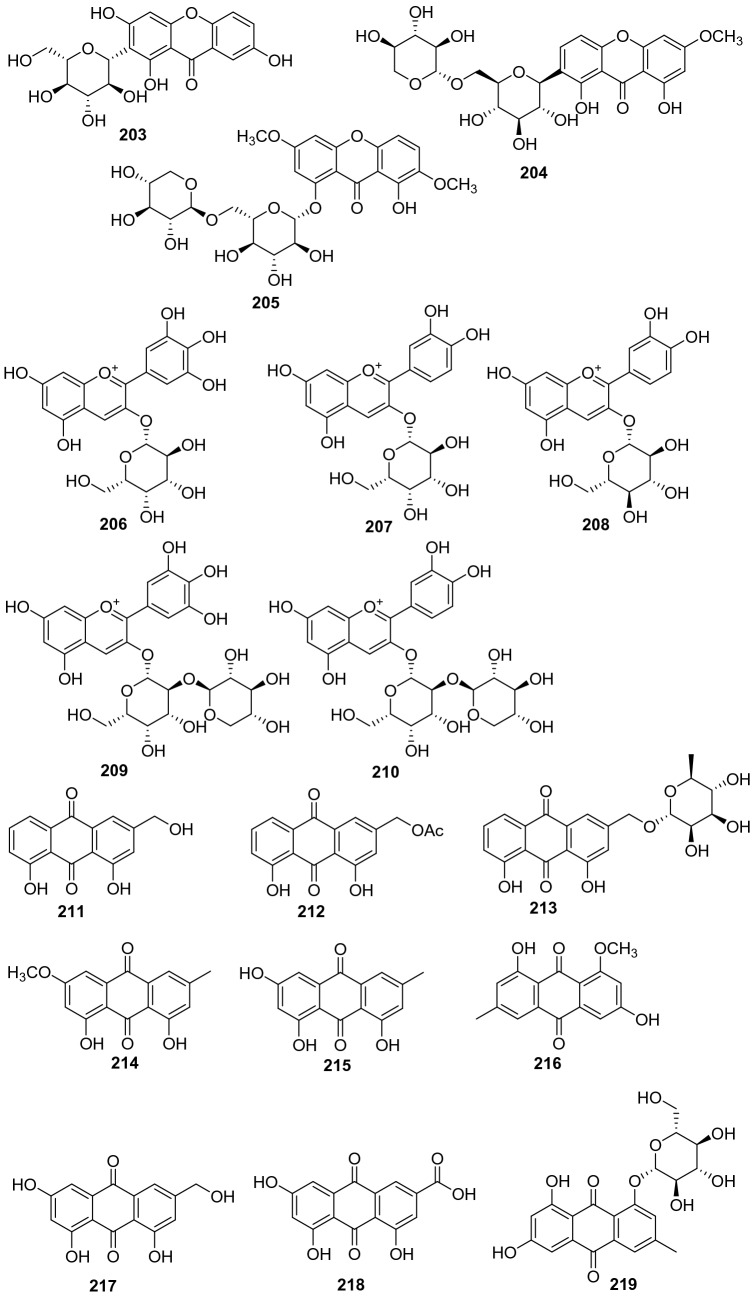
Structures of xanthones, isoflavones, and flavonols **203**‒**219** from *Actinidia* plants

Three xanthones were isolated from n-butyl alcohol fraction of *A. arguta* (Sieb. & Zucc) Planch. ex Miq and identified as 2-*β*-d-glu-1,3,7-trihydrogen xanthone **203**, 7-O-[*β*-d-xylose-(1 → 6)-*β*-d-glucopyranoside]-1,8-dihydroxy-3-methoxy xanthone **204**, and 1-O-[*β*-d-xylose-(1 → 6)-*β*-d-glucopyranside] -8-hydroxy-3,7-dimethoxy xanthone **205** (Fig. 9, Table 8). They were isolated from this plant for the first time [108]. Compound **203** showed extensive biological activities, including inhibiting *α*-Glycosidase, NO production inhibition and NF-*κ*B inhibition and PPAR activation [116, 117]. It has been demonstrated the inhibitory effects on NF-*κ*B transcriptional activation in HepG2 cells stimulated with TNF*α* with an IC_50_ value of 0.85 ± 0.07 μM, which was more potent than the positive control of sulfasalazine (IC_50_ = 0.9 μM) [118].

**Table 8:** Information of xanthones, anthocyanins, and emodins from *Actinidia* plants

No.	Compound name	Species Refs.	Part	Bioactivity Refs.
**203**	2-*β*-d-Glu-1,3,7-trihydrogen xanthone	*A. arguta* [108]	Roots	Inhibiting *α*-Glycosidase (in vitro) [116] NO inhibitory (in vitro) [117] NF-*κ*B Inhibition and PPAR Activation (in vitro) [118]
**204**	7-O-[*β*-d-Xylose-(1 → 6)-*β*-d-glucopyranoside]-1,8-dihydroxy-3-methoxy xanthone	*A. arguta* [108]	Roots	
**205**	1-O-[*β*-d-Xylose-(1 → 6)-*β*-d-glucopyranoside] -8-hydroxy-3,7-dimethoxy xanthone	*A. arguta* [108]	Roots	
**206**	Delphinidin 3-galactoside	*A. deliciosa* [119]	Fruits	Antioxidation (in vitro) [135]
**207**	Cyanidin 3-galactoside	*A. deliciosa* [119]	Fruits	
**208**	Cyanidin 3-glucoside	*A. deliciosa* [119]	Fruits	Anti-inflammatory (in vitro) [120, 121] Neuroprotective (in vivo) [122] Anti-cancer (in vitro) [123] Antioxidation (in vivo) [124]
**209**	Delphinidin 3-[2-(xylosyl)galactoside]	*A. deliciosa* [119]	Fruits	
**210**	Cyanidin 3-[2-(xylosyl)galactoside]	*A. deliciosa* [119]	Fruits	
**211**	Aloe-emodin	*A. deliciosa* [125]	Roots	Anti-inflammatory (in vitro) [126] Antifungal (in vitro) [127] Anticancer (in vitro) [128]
**212**	11-O-Acetyl-aloe-emodin	*A. deliciosa* [125]	Roots	
**213**	Aloe-emodin 11-O-*α*-l-rhamnopyranoside	*A. deliciosa* [125]	Roots	
**214**	Physcion (emodin-6-Me ether)	*A. chinensis* Planch [133]	Roots	
**215**	Emodin (frangala-emodin)	*A. chinensis* Planch [133]	Roots	Anti-inflammatory (in vivo* and *in vitro) [129] Neuroprotection (in vitro) [130] Anti-cardiovascular (in vitro) [131] Inhibiting *α*-Glucosidase (in vitro) [132]
**216**	Questin (emodin-8-Me ether)	*A. chinensis* Planch [133]	Roots	Hepatoprotection (in vitro) [134]
**217**	Citreorosein (*ω*-hydroxyemodin)	*A. chinensis* Planch [133]	Roots	
**218**	Emodic acid	*A. chinensis* Planch [133]	Roots	
**219**	Emodin-8-*β*-d-glucoside	*A. chinensis* Planch [133]	Roots	

#### Anthocyanins

Five anthocyanins were obtained from the flesh of larger fruit of *A. deliciosa* and *A. chinensis* and identified as delphinidin 3-galactoside **206**, cyanidin 3-galactoside **207**, cyanidin 3-glucoside **208**, delphinidin 3-[2-(xylosyl)galactoside] **209**, and cyanidin 3-[2-(xylosyl)galactoside] **210**, respectively (Fig. 9, Table 8) [119]. Cyanidin 3-glucoside **208** exhibited a wide range of pharmacological activities including anti-inflammatory, neuroprotective, anti-cancer, and antioxidant activities [120–124].

#### Emodins

A total of nine emodin derivatives were obtained (**211**‒**219**, Fig. 9, Table 8). Three emodin constituents were isolated from EtOAc fraction of the roots of *A. deliciosa* for the first time, and their structures were identified to be aloe-emodin **211**, 11-O-acetyl-aloe-emodin **212**, and aloe-emodin 11-O-*α*-l-rhamno -pyranoside **213** [125]. Compound **211** exhibited intriguing biological activities including inflammatory, antifungal, and anticancer activity [126–128]. Lipoxygenases (LOXs) are potential treatment targets in a variety of inflammatory conditions, enzyme kinetics showed that aloe emodin inhibited lipoxygenase competitively with an IC_50_ of 29.49 μM [126]. Compound **215** was reported to possess wide biological activities including anti-inflammatory, neuroprotection, anti-cardiovascular and *α*-glucosidase inhibitory activity [129–132]. It exhibited potent inhibition of α-glucosidase with an IC_50_ value of 19 ± 1 μM and lower cytotoxicity to the Caco-2 cell line [132].

#### Phenylpropionic Acids

**Fig. 10:**
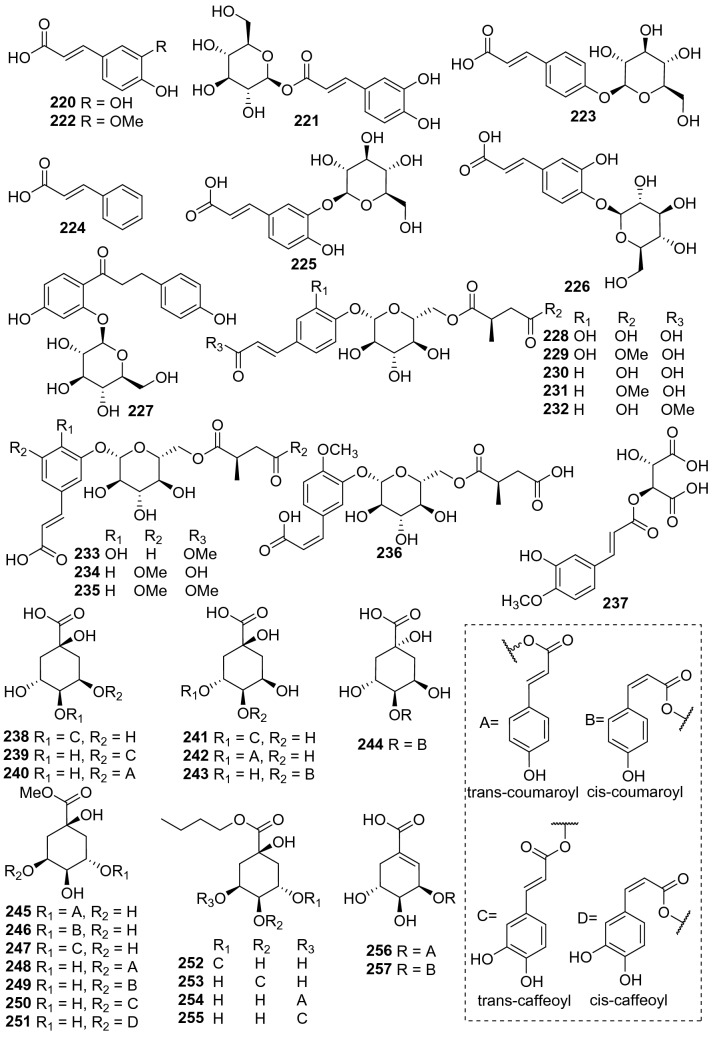
Structures of phenylpropionic acids **220**‒**257** from *Actinidia* plants

A total of 38 phenylpropionic acid derivatives have been identified from kiwifruit plants (**220**‒**257**. Fig. 10, Table 9), while most of them were glycosides or quinic acid derivatives. Phytochemical examination of the fruits of *A. arguta* led to the isolation of two organic acids including caffeic acid **220** and caffeoyl-*β*-d-glucopyranoside **221**, which were tested for their nitric oxide production inhibitory activity in LPS-stimulated RAW 264.7 cells and DPPH radical scavenging activities. Compared with positive control (L-NMMA), they were potently reduced nitric oxide productions and showed anti-oxidative activities [135]. Nine succinic acid derivatives (**228**‒**236**), eleven quinic acid (**245**‒**255**) derivatives and two shikimic acid derivatives (**256** and **257**) were isolated from the fruits of *A. arguta*. The NF-*κ*B transcriptional inhibitory activity of the compounds was evaluated using RAW 264.7 macrophages cells induced by lipopolysaccharide. Among the groups of different organic acid derivatives, the quinic acid derivatives inhibited NF-*κ*B transcriptional activity with an IC_50_ value of 4.0 μM [136].

**Table 9:** Information of phenylpropionic acids from *Actinidia* plants

No.	Compound name	Species Refs.	Part	Bioactivity Refs.
**220**	Caffeic acid	*A. arguta* [135]	Fruits	Anti-oxidation (in vitro) [135]
**221**	Caffeoyl-*β*-d-glucopyranoside	*A. arguta* [135]	Fruits	Anti-oxidation (in vitro) [135]
**222**	*Trans*-ferulic acid	*A. polygama* [110]	Aerial parts	
**223**	Coumaric acid 4-O-glucoside	*A. polygama* [110]	Aerial parts	
**224**	*Trans*-phydroxycinnamic acid	*A. chinensis* [137]	Roots	
**225**	Caffeic 3-O-*β*-d-glucopyranoside acid	*A. deliciosa* [89]	Pulps	
**226**	Caffeic 4-O-*β*-d-glucopyranoside acid	*A. deliciosa* [89]	Pulps	
**227**	4,4′-Dihydroxyl-dihydrochalcone-2′-O-*β*-d-glucopyranoside	*A. chinensis* Plach [52]	Roots	
**228**	Argutinoside A	*A. arguta* [136]	Fruits	
**229**	Argutinoside B	*A. arguta* [136]	Fruits	
**230**	Argutinoside C	*A. arguta* [136]	Fruits	
**231**	Argutinoside D	*A. arguta* [136]	Fruits	
**232**	Argutinoside E	*A. arguta* [136]	Fruits	
**233**	Argutinoside F	*A. arguta* [136]	Fruits	
**234**	Argutinoside G	*A. arguta* [136]	Fruits	
**235**	Argutinoside H	*A. arguta* [136]	Fruits	
**236**	Argutinoside I	*A. arguta* [136]	Fruits	
**237**	Fertaric acid	*A. arguta* [138]	Fruits	
**238**	Cryptochlorogenic acid	*A. chinensis* [137]	Roots	
**239**	Neochlorogenic acid	*A. chinensis* [137]	Roots	Anti-inflammatory (in vitro) [139] Antibacteria and antioxidation (in vitro) [140]
**240**	3-O-Coumaroylquinic acid	*A. chinensis* [137]	Roots	
**241**	Chlorogenic acid	*A. deliciosa* [89]	Pulp	Antitumor (in vitro) [141] Anti-inflammatory (in vitro) [142]
**242**	5- *Trans*-*p*-coumaroylmalic acid	*A. chinensis* Radix [50]	Roots	
**243**	5- *Cis*-*p*-coumaroylquinic acid	*A. chinensis* Radix [50]	Roots	
**244**	4-O- *Cis*-*p*-coumaroylquinic acid	*A. chinensis* Radix [50]	Roots	
**245**	3-O-*Trans*-*p*-coumaroyl quinic acid methyl ester	*A. arguta* [136]	Fruits	Inhibiting NF-*κ*B transcription (in vitro) [136]
**246**	3-O-*Cis*-*p*-coumaroyl quinic acid methyl ester	*A. arguta* [136]	Fruits	
**247**	3-O-*Trans*-*p*-caffeoyl quinic acid methyl ester	*A. arguta* [136]	Fruits	
**248**	5-O-*Trans*-*p*-coumaroyl quinic acid methyl ester	*A. arguta* [136]	Fruits	
**249**	5-O-*Cis*-*p*-coumaroyl quinic acid methyl ester	*A. arguta* [136]	Fruits	
**250**	5-O-*Trans*-*p*-caffeoyl quinic acid methyl ester	*A. arguta* [136]	Fruits	
**251**	5-O-*Cis*-*p*-caffeoyl quinic acid methyl ester	*A. arguta* [136]	Fruits	
**252**	3-O-*Trans*-*p*-caffeoyl quinic acid butyl ester	*A. arguta* [136]	Fruits	
**253**	4-O-*Trans*-*p*-caffeoyl quinic acid butyl ester	*A. arguta* [136]	Fruits	
**254**	5-O-*Trans*-*p*-coumaroyl quinic acid butyl ester	*A. arguta* [136]	Fruits	
**255**	5-O-*Trans*-*p*-caffeoyl quinic acid butyl ester	*A. arguta* [136]	Fruits	
**256**	3-O-*Trans*-*p*-coumaroyl shikimic acid	*A. arguta* [136]	Fruits	
**257**	3-O-*Cis*-*p*-coumaroyl shikimic acid	*A. arguta* [136]	Fruits	

#### Coumarins

**Fig. 11:**
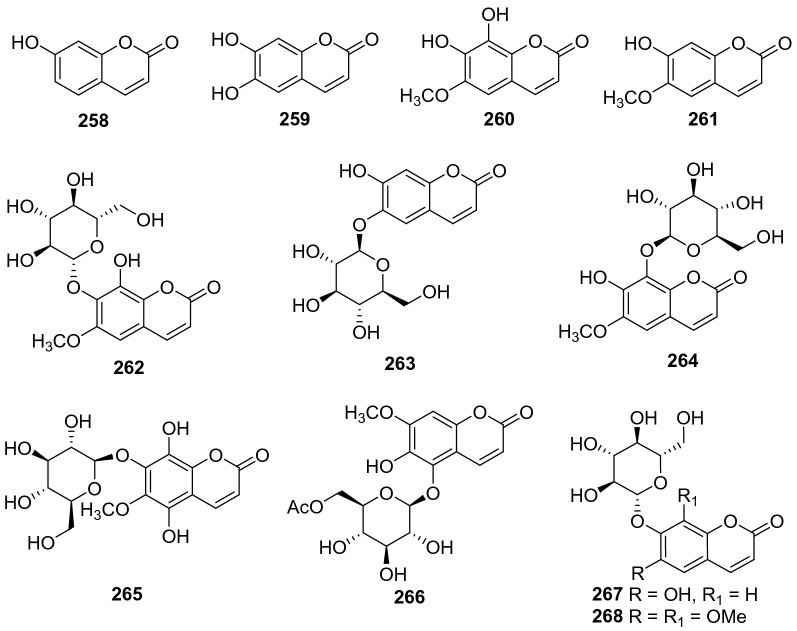
Structures of coumarins **258**‒**268** from *Actinidia* plants

Coumarins are rarely identified from kiwifruit plants, and only eleven members have been reported (**258**‒**267**, Fig. 11, Table 10). Umbelliferone **258** was obtained from the leaves of *A. polygama* (Sieb. et Zucc.) Miq [109]. A number of studies demonstrate the pharmacological properties of **258** including antitumor, anti-inflammatory, antioxidant, antidiabetic, and immunomodulatory activities [143–149]. It showed cytotoxicity against MCF-7 and MDA-MB-231 cell lines with IC_50_ values of 15.56 and 10.31 μM, respectively [148]. Phytochemical examination of the fruits of *A. arguta* led to the isolation of esculetin **259** [135]. Two coumarins were isolated from the roots of *A*. *deliciosa* and identified as fraxetin **260** and isoscopoletin **261** [150]. Compound **260** showed potent inhibition against lipopolysaccharide (LPS)-induced nitric oxide (NO) generation with an IC_50_ value of 10.11 ± 0.47 µM [151]. Esculin **263** and fraxin **264** were characterized from the stems and fruits of *A*. *deliciosa* (kiwifruit) and *A*. *chinensis* [152]. Compound **264** showed inhibitory activity towards HepG2 with an IC_50_ value of 14.71 μM [153].

**Table 10:** Information of coumarins from *Actinidia* plants

No.	Compound name	Species Refs.	Part	Bioactivity Refs.
**258**	Umbelliferone	*A. polygama* [109]	Leaves	Anti-tumor (in vitro) [143] Anti-inflammatory (in vitro) [144] Antioxidant (in vitro) [149] Antidiabetic (in vitro and in vivo) [146] Immunomodulatory(in vitro) [148]
**259**	Esculetin	*A. arguta* [135]	Fruits	Anti-tumor (in vitro) [154] Anti-oxidant and anti-inflammatory (in vivo) [155]
**260**	Fraxetin	*A*. *deliciosa* [150]	Roots	Anti-inflammatory (in vitro) [151] Anti-tumor (in vitro) [156]
**261**	Isoscopoletin	*A*. *deliciosa* [150]	Roots	Anti-inflammatory [151]
**262**	Isofraxoside	*A. chinensis* [137]	Roots	
**263**	Esculin	*A*. *deliciosa* [152]	Stems	
**264**	Fraxin	*A*. *chinensis* [152]	Fruits	Anti-inflammatory (in vivo) [157] Antitumor (in vitro) [153]
**265**	5-Hydroxy-6-methoxy-7-O-*β*-d- glycopyranosylcoumarin	*A. chinensis* Radix [50]	Roots	
**266**	6′-Acetoxy-8-*β*-d-glucopyranosyloxy-7- hydroxy-6-methoxy-coumarin	*A. chinensis* Radix [50]	Roots	
**267**	6-Hydroxy7-(*β*-d-glucopyranosyloxy) coumarin	*A*. *deliciosa* [89]	Peels	
**268**	6,8-Dimethoxy-7-(*β*-d-glucopyranosyloxy) coumarin	*A*. *deliciosa* [89]	Peels	

#### Lignans

**Fig. 12:**
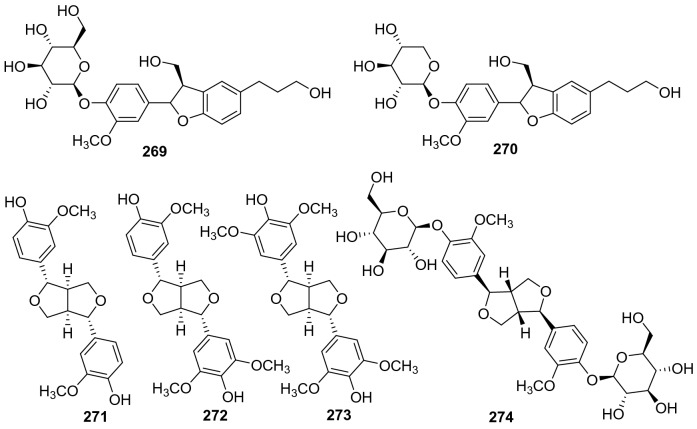
Structures of lignans **269**‒**274** from *Actinidia* plants

Lignans also had a narrow distribution in kiwi plants, only six members have been identified from *Actinidia* plants (**269**‒**274**, Fig. 12, Table 11). (+)-Pinoresinol **271**, (+)-medioresinol **272**, and (−)-syringaresinol **273** were partitioned from the fraction of the roots of *A. arguta* [53]. Compound **271** is a biologically active lignan and widely found in many dietary plants. It was reported to possess antifungal, anti-inflammatory, antioxidant, hypoglycemic, and antitumor activities [158–162]. A study on this compound suggested that **271** displayed significant inhibition of fMLP/CB-induced superoxide anion generation and elastase release, with an IC_50_ value of 1.3 ± 0.2 μg/mL [159]. The 50% ethanol extract of *A*. *arguta* showed strong inhibitory effect on *α*-glucosidase (32.6%), while a bio-guided isolation on the extract gave a bioactive compound pinoresinol diglucoside **274** [138].

**Table 11:** Information of of lignans from *Actinidia* plants

No.	Compound name	Species Refs.	Part		Cytotoxicity Refs.
**269**	Urolingoside	*A. polygama* [110]	Aerial parts		
**270**	4′-O-*β*-Dxylopyranoside	*A. chinensis* Radix [50]		Roots	
**271**	(+)-Pinoresinol	*A. arguta* [53]	Roots		Antifungal (in vitro) [158] Anti-inflammatory (in vitro) [159] Antioxidation (in vitro) [160] Hypoglycemic (in vitro) [161] Anti-tumor (in vitro) [162]
**272**	(+)-Medioresinol	*A. arguta* [53]	Roots		
**273**	(−)-Syringaresinol	*A. arguta* [53]	Roots		
**274**	Pinoresinol diglucoside	*A*. *arguta* [138]	Leaves		Inhibiting *α*-glucosidase (in vitro) [138]

#### Simple Phenols

**Fig. 13:**
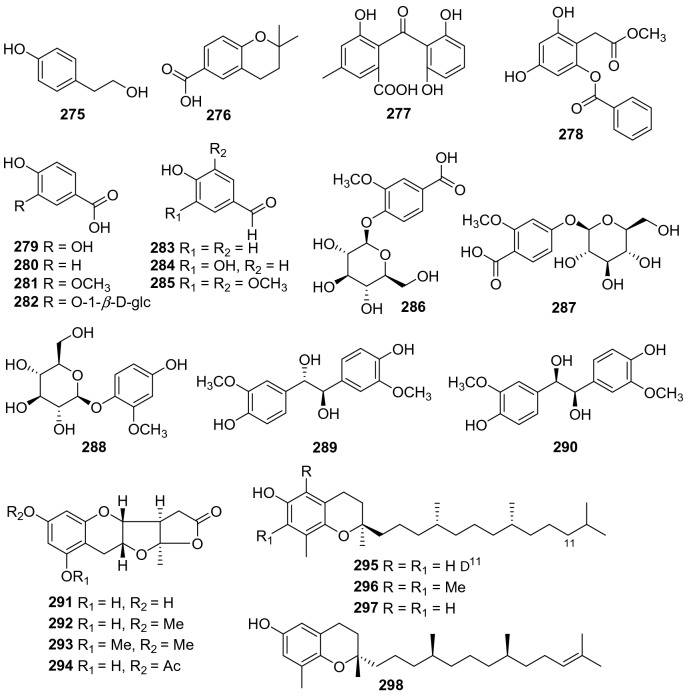
Structures of simple phenols **275**‒**298** from *Actinidia* plants

Simple benzene derivatives including glycosides and isoprenylated benzene products from *Actinidia* plants were collected (**275**‒**298**, Fig. 13, Table 12). Phytochemical examination of the fruits of *A. arguta* led to the isolation of protocatechuic acid **279** [135]. It showed anti-inflammatory [163], antioxidant [163], neuroprotective [164], and anti-proliferative activities [165]. Protocatechuic acid exhibited significant (p < 0.05) anti-inflammatory (83% and 88% inhibition for egg-albumin induced and xylene induced oedema, respectively), analgesic (56% inhibition and 22 s of pain suppression for acetic acid-induced and hot plate-induced pain, respectively), and antioxidant effects (97% inhibition and absorbance of 2.516 at 100 μg/mL for DPPH and FRAP assay, respectively) in the models [166]. Extraction of leaf tissue from the golden-fleshed kiwifruit cultivar *A. chinensis* “Hort16A” expressing genotype-resistance against the fungus *Botrytis cinerea*, a new phenolic compound, 3,5-dihydroxy-2-(methoxycarbonylmethyl)phenyl 3,4-dihydroxybenzoate **278** was therefore obtained [167].

**Table 12:** Information of simple phenols from *Actinidia* plants

No.	Compound name	Species Refs.	Part	Bioactivity Refs.
**275**	Tyrosol	*A. arguta* [168]	Roots	Anti-​inflammatory (in vitro) [151]
**276**	2,2-Dimethyl-6-chromancarboxylic acid	*A*. *deliciosa* [150]	Roots	
**277**	Monodictyphenone	*A. chinensis* Radix [50]	Roots	Inhibiting protein tyrosine phosphatase (in vitro) [169]
**278**	3,5-Dihydroxy-2-(methoxycarbonyl methyl) phenyl 3,4-dihydroxybenzoate	*A. chinensis* [167]	Leaves	
**279**	Protocatechuic acid	*A. arguta* [135]	Fruits	Anti-inflammatory and antioxidant (in vitro and in vivo) [163] Neuroprotective (in vitro) [164] Anti-proliferative (in vitro) [165]
**280**	4-Hydroxy benzoic acid	*A. arguta* [92]	Roots	
**281**	Vanillic acid	*A*. *deliciosa* [150]	Roots	
**282**	4-(*β*-d-Glucopyranosyloxy)-3-hydroxybenzoic acid	*A. chinensis* Radix [50]	Roots	
**283**	*p*-Hydroxybenzaldehyde	*A. chinensis* Radix [50]	Roots	
**284**	Tachioside	*A*. *deliciosa* [150]	Roots	
**285**	Syringaldehyde	*A. chinensis* Planch [105]	Roots	Anti-inflammatory (in vitro) [170]
**286**	Vanillic acid 4-O-*β*-d-glucopyranoside	*A*. *deliciosa* [150]	Roots	
**287**	Protocatechualdehyde	*A. chinensis* [171]	Roots	Antioxidant and anti-inflammatory (in vitro) [172]
**288**	4-Hydroxy-2-methoxyphenyl-*β*-d-glucopyranoside	*A. macrosperma* [66]	Roots	
**289**	Erythro-1,2-bis-(4-hydroxy-3-methoxyphenyl)-1,3-propanediol	*A. chinensis* [171]	Roots	
**290**	Threo-1,2-bis-(4-hydroxy-3-methoxyphenyl)-1,3-propanediol	*A. chinensis* [171]	Roots	
**291**	Planchols A	*A. chinensis* Planch [94]	Roots	Against P-388 and A-549 cell lines (in vitro) [94]
**292**	Planchols B	*A. chinensis* Planch [94]	Roots	Against P-388 and A-549 cell lines (in vitro) [94]
**293**	Planchols C	*A. chinensis* Planch [94]	Roots	Against P-388 and A-549 cell lines (in vitro) [94]
**294**	Planchols D	*A. chinensis* Planch [94]	Roots	Against P-388 and A-549 cell lines (in vitro) [94]
**295**	2,8-Dimethyl-2-(4,8,12-trimethyltridec-11-enyl)chroman-6-ol	*A*. *deliciosa* [89]	Peels	
**296**	*α*-Tocopherol	*A*. *deliciosa* [89]	Peels	
**297**	*δ*-Tocopherol	*A*. *deliciosa* [89]	Peels	
**298**	2,8-Dimethyl-2-(4,8,12-trimethyltridec-11-enyl)chroman-6-ol	*A. chinensis* [173]	Peels	

Four novel skeleton phenolic compounds planchols A‒D (**291**‒**294**) were isolated from the roots of *A. chinensis* Planch. Their structures were elucidated by spectroscopic analysis and chemical evidence. The structure of **291** was further confirmed by the single-crystal X-ray diffraction. Moreover, it was found that **291** and **292** showed remarkable cytotoxic activity against P-388 with IC_50_ of 2.50 and 3.85 μM, respectively, and against A-549 with IC_50_ of 1.42 and 2.88 μM, respectively [94].

### Miscellaneous

**Fig. 14:**
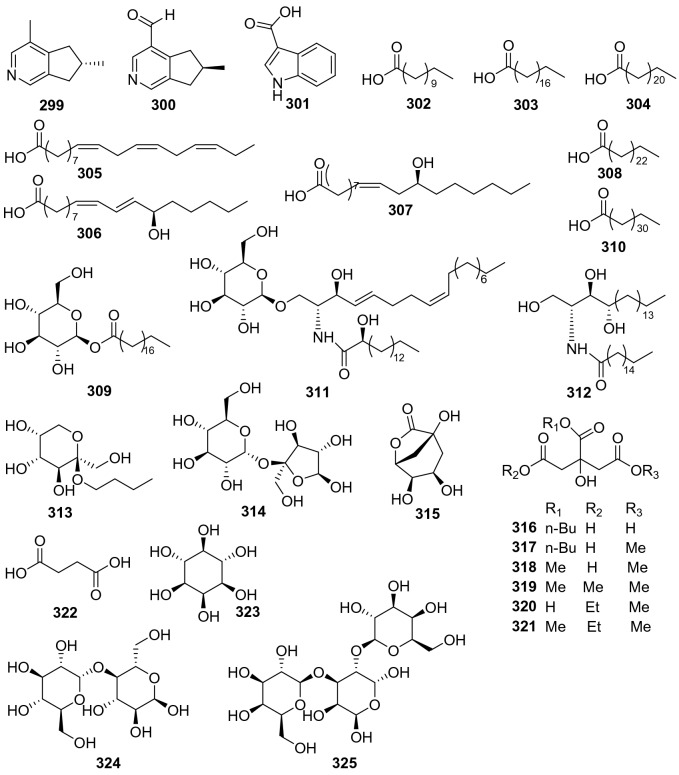
Structures of other molecules **299**‒**325** from *Actinidia* plants

Three alkaloids (**299**‒**301**), eleven fatty acids and derivatives (**302**‒**312**), and other thirteen small molecules (**313**‒**325**) were obtained from *Actinidia* plants (Fig. 14, Table 13). Actinidine **299** and boschniakine **300** were isolated from the leaves and galls of *A*. *polygama* and also isolated from *A. arguta* which might be converted from iridoids [174, 175]. A bioassay-guided fractionation of the fruits of *A. polygama* led to the separation and identification of a polyunsaturated fatty acid, *α*-linolenic acid (ALA) **305** [176]. This compound was found to possess a broad biological properties including anti-inflammatory [177], anti-tumor [178], anti-hyperlipidemic [179], anti-diabetic [180], and anti-fungal [181] activities. By a bio-guided fractionation, a ceramide namely actinidiamide **312** was identified as an anti-inflammatory component from the EtOAc fraction of *A. polygama* Max. It potently inhibited nitric oxide production (30.6% inhibition at 1 μg/mL) in lipopolysaccharide (LPS)-stimulated RAW264.7 cells and *β*-hexosaminidase release (91.8% inhibition at 1 μg/mL) in IgE-sentized RBL-2H3 cells [182].

**Table 13:** Information other molecules from *Actinidia* plants

No.	Compound name	Species Refs.	Part	Bioactivity Refs.
**299**	Actinidine	*A*. *polygama* [174]	Leaves and galls	Anti-obesity (in vivo) [183]
**300**	Boschniakine	*A. arguta* [175]		
**301**	Indole-3-carboxylic acid	*A. chinensis* Planch [184]	Roots	
**302**	Undecanoic acid	*A. deliciosa* [185]	Roots	Anti-inflammatory (in vivo) [186]
**303**	n-Stearic acid	*A. deliciosa* [150]	Roots	
**304**	Tetracosanoic acid	*A. eriantha* Benth [41]	Roots	
**305**	*α*-Linolenic acid	*A. polygama* [176]	Fruits	Anti-inflammatory (in vivo) [177] Anti-tumor (in vivo) [178] Anti-hyperlipidemic (in vivo) [179] Anti-diabetic (in vivo) [180] Anti-fungal (in vitro) [181]
**306**	(9*Z*,11*E*)-13-Hydroxy-9,11-octadecadienoic acid	*A. chinensis* Radix [50]	Roots	
**307**	Ricinoleic acid	*A. chinensis* Radix [50]	Roots	
**308**	Lignoceric acid	*A. chinensis* Planch [187]	Roots	
**309**	Stearyl-*β*-d-glucopyranoside	*A. chinensis* Planch [187]	Roots	
**310**	Dotriacontanic acid	*A. chinensis* Planch [52]	Roots	
**311**	Sphingolipid	*A. chinensis* Planch [52]	Roots	
**312**	Actinidiamide	*A. polygama* [182]	Fruits	Anti-inflammatory (in vitro) [182]
**313**	*n*-Butyl-O-*β*-d-fructopyranoside	*A. deliciosa* [188]	Roots	
**314**	Sucrose	*A. chinensis* Planch [187]	Roots	
**315**	*γ*-Quinide	*A. chinensis* Planch [187]	Roots	
**316**	1*-*Methyl*-*5*-*ethyl citrate	*A. arguta* [136]	Fruits	
**317**	1,6-Dimethyl citrate	*A. arguta* [136]	Fruits	
**318**	1,5,6-Trimethyl citrate	*A. arguta* [136]	Fruits	
**319**	1,6-Dimethyl-5-ethyl citrate	*A. arguta* [136]	Fruits	
**320**	6-Butyl citrate	*A. arguta* [136]	Fruits	
**321**	1-Methyl-6-butyl citrate	*A. arguta* [136]	Fruits	
**322**	Succinic acid	*A*. *kolomikta* [189]	Leaves	
**323**	Meso-inositol	*A*. *kolomikta* [189]	Leaves	
**324**	Maltose	*A*. *kolomikta* [189]	Leaves	
*325*	*α*-Kolomiktriose	*A*. *kolomikta* [190]	Roots	

In summary, this review focused on the biological components and related pharmacological activities of various parts of *Actinidia* plants, including triterpenoids, steroids, flavonoids, catechins, coumarins, lignans, phenols, and other small organic molecules. A total of 325 molecules have been collected in this review. Most of the active molecules are derived from the roots of *Actinidia* plants, while triterpenes and flavonoids are the most important types regardless of the number of compounds and their biological activity significance. The stems, leaves, fruit galls, and other parts of kiwi are mainly rich in flavonoids, phenylpropionic acids, and other small molecule compounds. Currently, these chemical components are not structurally novel. In addition, there are few in-depth researches on pharmacological activities of the bioactive compounds. Therefore, research on the chemical constituents of *Actinidia* plants is still promising. We hope that this review can provide positive information for the further exploration of the chemical components and their biological activities of *Actinidia* plants.
